# Slowly but surely: Exposure of communities and infrastructure to subsidence on the US east coast

**DOI:** 10.1093/pnasnexus/pgad426

**Published:** 2024-01-02

**Authors:** Leonard O Ohenhen, Manoochehr Shirzaei, Patrick L Barnard

**Affiliations:** Department of Geosciences, Virginia Tech, Blacksburg, VA 204061, USA; Virginia Tech National Security Institute, Virginia Tech, Blacksburg, VA 24061, USA; Department of Geosciences, Virginia Tech, Blacksburg, VA 204061, USA; Virginia Tech National Security Institute, Virginia Tech, Blacksburg, VA 24061, USA; Institute for Water, Environment and Health, United Nations University, Hamilton, ON L8P 0A1, CA; U.S. Geological Survey, Pacific Coastal and Marine Science Center, Santa Cruz, CA 95060, USA

**Keywords:** coastal hazard, land subsidence, infrastructure hazards, exposure

## Abstract

Coastal communities are vulnerable to multihazards, which are exacerbated by land subsidence. On the US east coast, the high density of population and assets amplifies the region's exposure to coastal hazards. We utilized measurements of vertical land motion rates obtained from analysis of radar datasets to evaluate the subsidence-hazard exposure to population, assets, and infrastructure systems/facilities along the US east coast. Here, we show that 2,000 to 74,000 km^2^ land area, 1.2 to 14 million people, 476,000 to 6.3 million properties, and >50% of infrastructures in major cities such as New York, Baltimore, and Norfolk are exposed to subsidence rates between 1 and 2 mm per year. Additionally, our analysis indicates a notable trend: as subsidence rates increase, the extent of area exposed to these hazards correspondingly decreases. Our analysis has far-reaching implications for community and infrastructure resilience planning, emphasizing the need for a targeted approach in transitioning from reactive to proactive hazard mitigation strategies in the era of climate change.

**Significance Statement**

By quantifying the exposure to subsidence hazards for coastal communities and infrastructure, this study finds that subsidence rates of 2 mm per year affects a maximum of 2.1 million people, 867,000 properties (median exposure), and significant infrastructure on the East Coast of the United States. This hazard is a major threat to metropolitan cities such as New York, Baltimore, and Norfolk, whose populations and properties intersect directly with the rising seas. Our study provides important quantitative data for coastal disaster resilience planning.

## Introduction

Coastal regions, where most megacities are located, are on the front lines of climate change impacts and associated uncertainties (1–4). The coincidence of population migration toward low-elevation coastal areas and continued accelerating sea-level rise (SLR) will increase the future vulnerability of coastal communities worldwide (5–7). The impact of SLR-amplified hazards on coastal communities, such as flooding and erosion, dominates in global climate change discussions (1, 6, 8), with other coastal hazards, such as land subsidence (the lowering of land elevation) relegated to the background. Land subsidence, however, is a pernicious and growing problem on a global scale with more immediate hazards to coastal areas and often presents more pressing and localized challenges (9–15). In many nations, land subsidence barely registers as an issue of public policy (11). In nations, where the adverse effects of subsidence are recognized, the slow, gradual, and unapparent land sinking motion explains the lack of urgent policy interventions and subsidence governance (11, 15). The resulting delayed response increases the exposure of coastal residents, especially in light of the yearly elevation gain in sea levels due to climate change and elevation loss due to subsidence. Recent considerations of the combined effect of SLR and subsidence indicate that subsidence increases the threat to coastal communities from SLR and may even triple estimates of potential flooding areas over the next few decades (3, 16, 17).

**Fig. 1.**
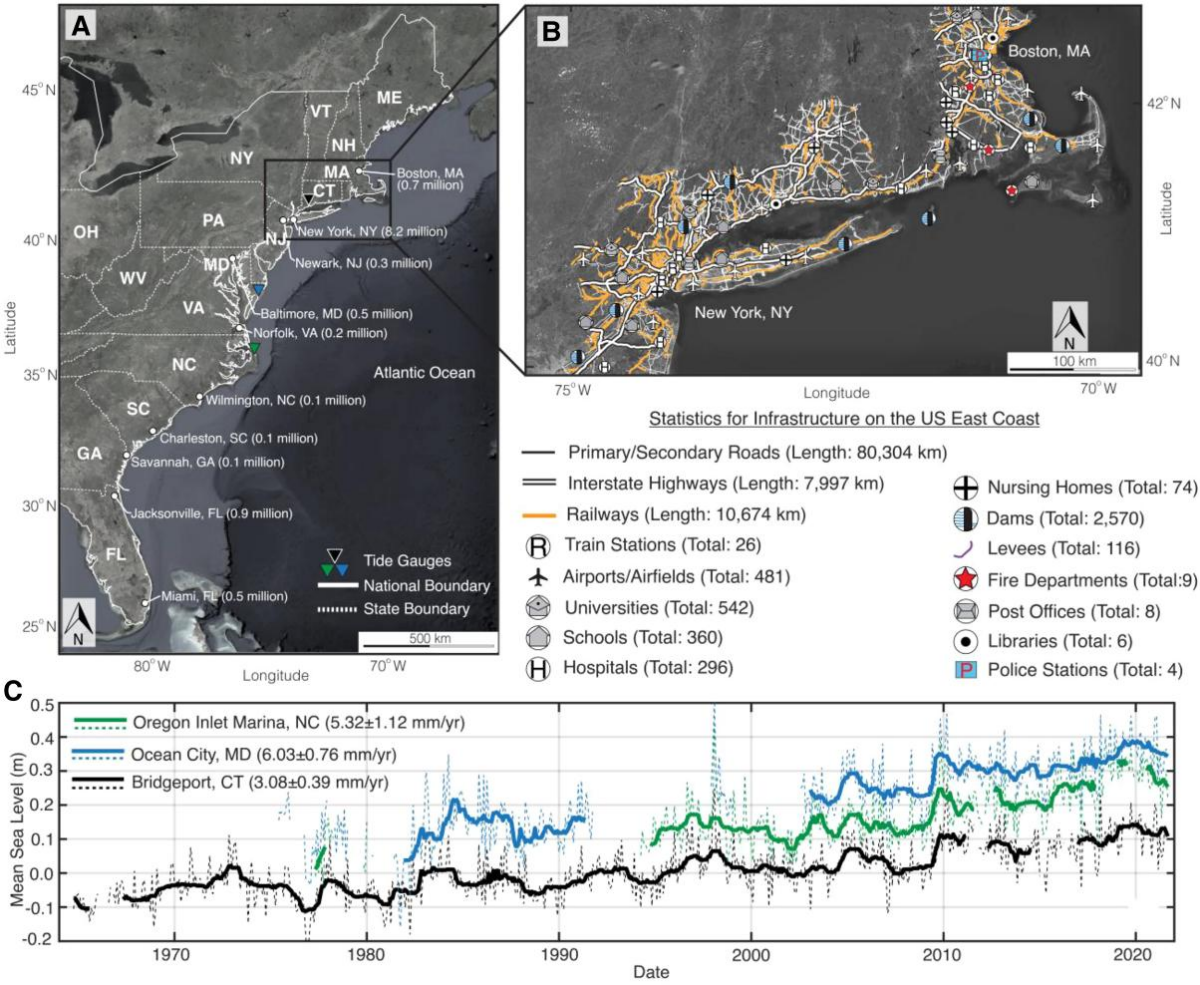
Population, infrastructure and SLR on the US east coast. A) Population distribution in major cities on the US east coast. The population data are the 2020 population estimate from the US census bureau (https://www.census.gov/geographies/mapping-files/time-series/geo/tiger-data.2010.html). The rectangle indicates extent of the close-up area shown in (B). B) Close-up view of New England showing Infrastructure systems/facilities on the US east coast. The summary table beneath (B) represents statistics for all infrastructure facilities analyzed in this study. Background Images in (A) and (B) are from Google, Earthstar. C) Time series of monthly (dashed line) and annual (solid line) mean sea level for tide gauge stations shown as inverted triangles in (A) (21). The time series have been offset for visual clarity.

On the East Coast of the United States, the high density of population and infrastructure networks (Fig. 1A, B), coupled with SLR (Fig. 1C) and land subsidence hazard, increases the exposure of the population, properties, and assets in the region (13, 18). The functionality, mobility, social comfort, and economic growth and development of society depend on civil infrastructure networks (19). Aging stock, extreme weather events, and differential land subsidence negatively impact infrastructure networks' safety (19). The 2021 American Society of Civil Engineers (ASCE) report card for airports, schools, roads, bridges, dams, and levees in the United States found that these infrastructures were in “mediocre” or “poor” condition, with only railways in “good” condition (20). A similar infrastructure assessment in 14 coastal states on the US east coast (Florida [FL] to Maine) indicates an overall “poor” condition. The ASCE report estimates that US$786 billion, US$125 billion, and US$45.2 billion are needed for the backlog of roads, bridges, and railways maintenance. This complacency toward maintenance in a high-hazard-prone coastal area increases the susceptibility of infrastructure to failure.

Infrastructure failure has major environmental repercussions and can result in significant fatalities. For example, in August 2005, Hurricane Katrina caused the breaching of the New Orleans levee system, resulting in widespread flooding, extensive damage to the city, and the loss of over 1,500 lives (22, 23). The 2013 collapse of the eight-story Rana Plaza Savar, Dhaka, Bangladesh, resulted in >1,100 deaths (24). On 2018 August 14, the Morandi bridge in Genoa, Italy collapsed over the Polcevera river, causing 43 deaths (25). The July 2021 12-story building collapse in Miami, FL, claimed the lives of 98 people (26). These high-consequence events in coastal areas are unfortunate reminders of the overwhelming and devastating consequences of infrastructure failure. Settlement (or subsidence) in an area affects the integrity of existing structures and increases the likelihood of failure (27–30). For some structures, unless foundations reach the bedrock, they are more susceptible to settlement and subsidence hazards, which can lead to eventual failure if not properly managed (31). Thus, frequently monitoring the conditions of infrastructure and associated hazards is essential for resilience assessment by individual asset owners, engineers, and policymakers (32).

The exploitation of deformation signals from interferometric synthetic aperture radar (InSAR) has proved effective in assessing at-risk populations (9, 33) and monitoring infrastructure resilience, e.g. roads, railways, bridges, airports, dams, and levees (19, 25, 34–38). Here, we investigate the exposure of communities, assets, and 14 infrastructure systems/facilities (roads, railways, dams, levees, train stations, airports/airfields, universities, schools, hospitals, nursing homes, fire departments, post offices, libraries, and police stations) to subsidence hazards within 100 km inland of the US east coast. In this study, we define exposure to subsidence in terms of the area, population, properties, and infrastructure interacting directly with the sinking land on the US east coast, for varying magnitudes of subsidence rate (see Materials and methods). The subsidence exposure analysis is critical for coastal hazard (e.g. flooding) mitigation and infrastructure resilience development on the US east coast and for linking subsidence to the socioeconomic consequences of relative SLR.

## Results

### Land subsidence along the US east coast

To characterize the land deformation along the US east coast, we leverage the ∼50 m resolution vertical land motion (VLM) datasets from 2007 to 2020 published by the United States Geological Survey (USGS) (39, 40) and Ohenhen et al. (41) (Fig. S1A). The dataset was produced by combining thousands of images comprising 96 SAR frames from two satellites (Sentinel-1 A/B and ALOS-1) with measurements from 162 global navigation satellite system (GNSS) stations (Fig. S2). Using a χ^2^ goodness of fit test, we performed a statistical hypothesis testing to account for the impact of measurement errors or model deficiencies on the quality of the estimated VLM rates (Fig. S1B; see Materials and methods). The hypothesis testing applied to the dataset ensured the robustness of our subsequent exposure analysis.

**Fig. 2.**
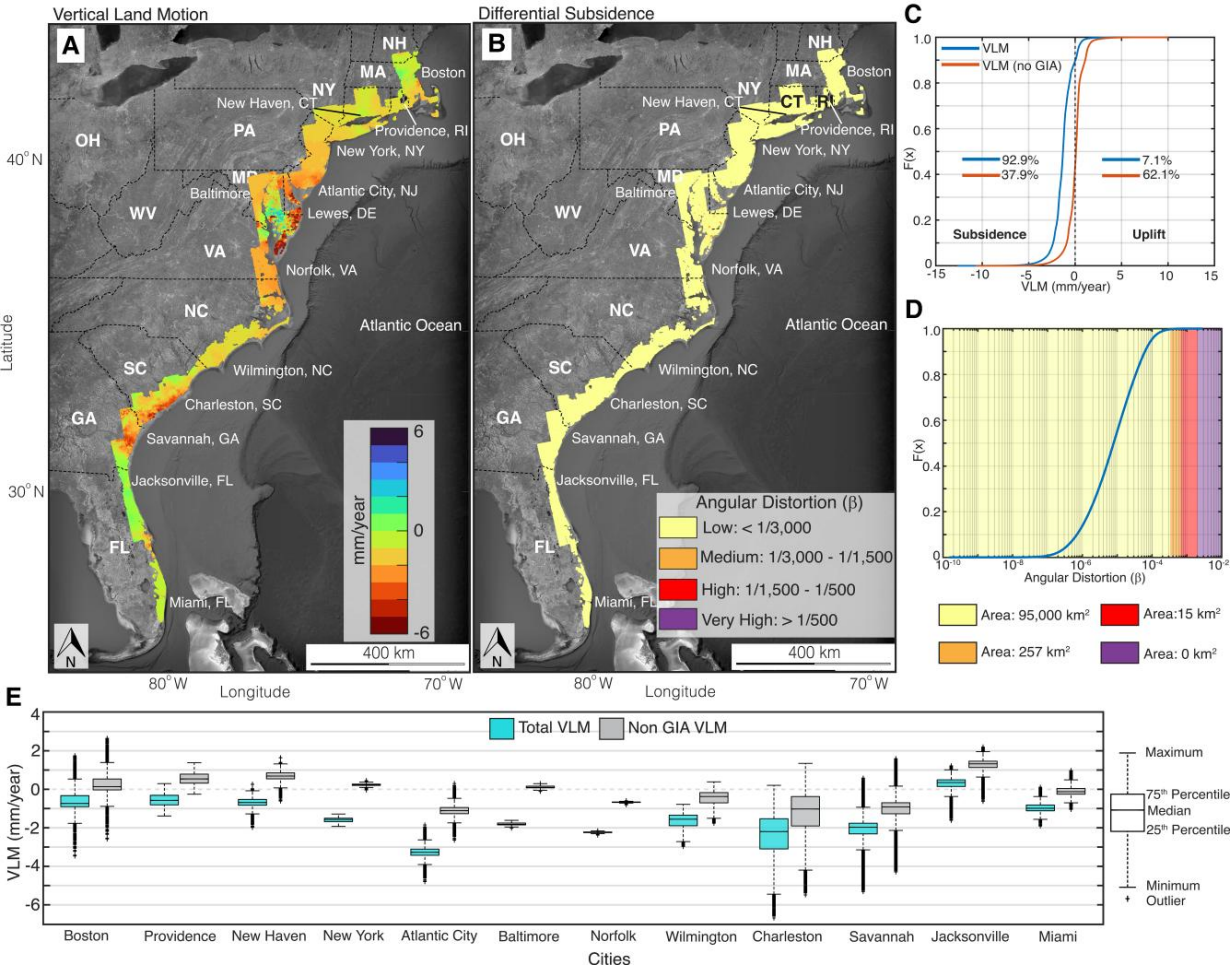
Vertical land motion (VLM) along the US east coast. A) Updated VLM rate for the US east coast from this study. This map includes ∼25 million pixels at ∼50 m resolution. B) Spatial distribution of angular distortion (*β*) map. Background Images in (A) and (B) are from Google, Earthstar. C) Empirical cumulative distribution function for VLM and VLM without GIA. The GIA data from the ICE-6G-D model (42). D) Empirical cumulative distribution function for *β*. The area for each *β* category is estimated using a regular grid of 50 m. E) Distribution of land subsidence for 12 metropolitan cities on the US east coast: Boston (MA), Providence (RI), New Haven (CT), New York (NY), Atlantic City (NJ), Baltimore (MD), Norfolk (VA), Wilmington (NC), Charleston (SC), Savannah (GA), Jacksonville (FL), and Miami (FL). The locations of the cities are highlighted in (A).

Figure 2A shows the updated VLM from this study, with negative values indicative of land subsidence and positive values indicating uplift. Hereafter, we quote VLM values with the respected signs, while we only report the absolute values for subsidence (i.e. negative VLM). Given the standard deviations, 78 to 99% of the obtained InSAR pixels show subsidence with a median rate of 1.3 ± 0.5 mm per year (Figs. S3 and S4). The maximum subsidence rate across the US east coast exceeds 10 mm per year (Fig. S3) and varies with rates up to 13 mm per year (Fig. S3). We highlight 12 metropolitan cities affected by spatially variable land subsidence (Fig. 2E): Boston (Massachusetts [MA]), Providence (Rhode Island [RI]), New Haven (Connecticut [CT]), New York (New York [NY]), Atlantic City (New Jersey [NJ]), Baltimore (Maryland [MD]), Norfolk (Virginia [VA]), Wilmington (North Carolina [NC]), Charleston (South Carolina [SC]), Jacksonville (FL), and Miami (FL). Note the different ranges of rates of each city illustrated by the box plots and corresponding outliers. We observe subsidence in most cities, with rates exceeding 1 mm per year in some areas. Notably, several areas in Atlantic City, Savannah, and Charleston are subsiding with rates faster than 4 mm per year (Fig. 2E).

To analyze the factors contributing to VLM along the US east coast, we employed the glacial isostatic adjustment (GIA) ICE-6G-D model (42) to estimate the GIA contributions at the InSAR pixels and excluded its effect from the observed VLM (Fig. S5A). In the absence of the long wavelength GIA signals, the corresponding VLM map is dominated by non-GIA signals, including the contribution of anthropogenic influences (e.g. groundwater extraction and sediment compaction). The VLM from the GIA model displays a persistent subsidence signal along the US east coast, with particularly high subsidence rates of ∼3 mm per year observed in NJ and Delaware (DE) (Fig. S5B). Without the GIA effects, the subsidence signal in the VLM map decreases by 55% (Fig. 2C), suggesting that GIA effects predominate and control the observed broad-scale subsidence along the US east coast. The median non-GIA VLM rates show an apparent shift toward uplift (VLM > 0 mm per year) in half of the 12 highlighted major cities. However, cities such as Boston, Atlantic City, Charleston, and Savannah show non-GIA subsidence rates of 1 mm per year in some areas (Fig. 2E).

### Differential subsidence along the US east coast

Differential subsidence, the uneven sinking of the land, poses a significant hazard to urban infrastructure, including buildings, roads, and other infrastructure facilities. The hazard associated with differential subsidence is due to angular distortion (*β*) caused by strain changes between two adjacent points, which is commonly employed in geotechnical engineering to assess the severity of damage caused by subsidence/differential settlement (e.g. refs. 29, 30, 43, 44). We employed the angular distortion (*β*) to assess the hazard of differential subsidence for the US east coast (Eq. 7; see Materials and methods). Following Cigna and Tapete (29), we classified the hazard into four categories based on the angular distortion (*β*) values: low (β<1/3,000), medium (1/3,000≤β<1/1,500), high (1/1,500≤β≤1/500), and very high (β>1/500), indicative of increasing hazard severity.

The angular distortion map (Fig. 2B) shows that >99.5% of the area (95,000 km^2^) are classified as low-hazard zones for the time scale of InSAR period (2007–2020). We find no area with β>1/500 (very high-hazard zones), which is the recommended threshold for severe damage to urban infrastructure (27, 43). However, the presence of medium and high *β* zones in a combined area of 290 km^2^, predominantly around the Chesapeake Bay area, where high subsidence and uplift signals are noted. These medium and high-hazard areas suggest uneven settlement in these regions and should be the focus of frequent monitoring, particularly in urban areas. Analysis of the differential subsidence for urban areas on the US east coast show that *β* is low for the 12 highlighted major cities, with only Boston having a medium *β* value in 0.05 km^2^ area (Table S1).

### County-specific subsidence exposure analysis

**Fig. 3.**
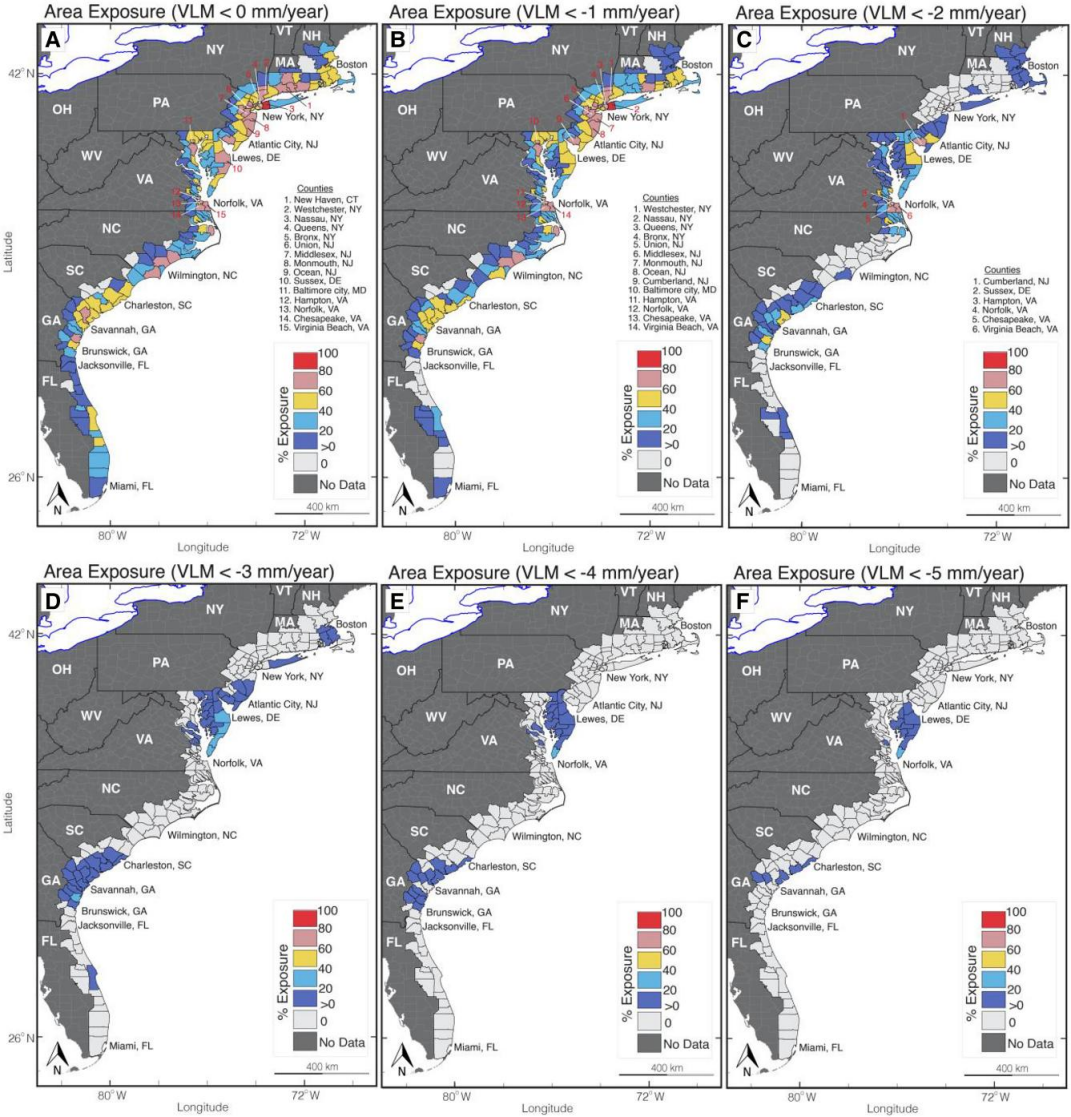
Subsidence area exposure for counties on the US east coast. A) Percentage of county’s land area affected by VLM < 0 mm per year. B) Percentage of county’s land area affected by VLM < −1 mm per year. C) Percentage of county’s land area affected by VLM < −2 mm per year. D) Percentage of county’s land area affected by VLM < −3 mm per year. E) Percentage of county’s land area affected by VLM < −4 mm per year. F) Percentage of county’s land area affected by VLM < −5 mm per year. Note that this figure considers the median bound (VLM only), see Figs. S6 and S7 for the lower and upper bounds (VLM ± standard deviation). The shape outline in (A–F) defines the extent of each county. Important counties are numbered in (A) to (F). Table 1 summarizes the number of counties, total land area in km^2^, population, and properties exposed to subsidence. A summary table of all counties and land area exposure is provided in Tables S3–S5.

To determine the area affected by subsidence on a by-county basis, we calculated the percent area in each county affected by subsidence using Eq. (8) and evaluated the area exposure for different subsidence hazard severity (see Materials and methods). This assessment incorporates the standard deviation values from the VLM, providing specific lower, median, and upper bounds for the subsidence exposure analysis in each county (see Materials and methods). Figures 3 (median), S6 (lower bound), and S7 (upper bound) show the percent area exposed to subsidence hazards in coastal counties (172 counties overlapping with SAR pixels) on the US east coast, while Table 1 details the total land area, population, and property exposure. We only estimate population and property exposure for counties with high area exposure (>60%).

**Table 1.** Subsidence exposure for counties, population, and properties on the US east coast.

VLM (mm per year)	Area exposure (%)	Number of counties	Total land area (×10^3^ km^2^)	Population exposure (million)	Property exposure (million)
<0	>0–20	47, 39, 34	5.6, 4.8, 4.6	NA	NA
	20–40	50, 47, 44	21.2, 17.1, 21.1	NA	NA
	40–60	32, 46, 53	17.4, 24.7, 33.1	NA	NA
	60–80	24, 30, 31	14.6, 20.3, 22.0	13.0, 14.4, 15.1	5.1, 5.8, 6.0
	>80	1	0.6	1.4	0.5
<−1	>0–20	70, 51, 40	7.7, 4.6, 5.0	NA	NA
	20–40	35, 41, 43	9.6, 13.9, 19.3	NA	NA
	40–60	15, 39, 49	5.6, 20.7, 28.6	NA	NA
	60–80	18, 23, 30	7.1, 12.5, 20.7	11.8, 12.1, 14.4	4.1, 4.7, 5.8
	>80	0, 1, 1	0.0, 0.6, 0.6	0, 1.4, 1.4	0, 0.5, 0.5
<−2	>0–20	53, 59, 80	1.5, 3.6, 6.5	NA	NA
	20–40	3, 20, 33	0.6, 5.3, 11.8	NA	NA
	40–60	0, 10, 30	0.0, 4.4, 15.5	NA	NA
	60–80	0, 6, 9	0.0, 2.0, 5.6	0, 1.2, 2.4	0, 0.5, 1.2
	>80	0	0.0	0	0
<−3	>0–20	13, 43, 64	0.3, 1.7, 4.0	NA	NA
	20–40	1, 5, 12	0.2, 1.8, 4.1	NA	NA
	40–60	0, 0, 9	0.0, 0.0, 4.8	NA	NA
	60–80	0, 0, 1	0.0, 0.0, 1.5	0, 0, 0.2	0, 0, 0.1
	>80	0	0	0	0
<−4	>0–20	8, 28, 52	0.2, 1.1, 3.3	NA	NA
	20–40	1, 1, 5	0.1, 0.2, 1.8	NA	NA
	40–60	0, 0, 2	0.0, 0.0, 1.9	NA	NA
	60–80	0	0.0	0	0
	>80	0	0.0	0	0
<−5	>0–20	7, 13, 38	0.1, 0.4, 1.3	NA	NA
	20–40	0, 1, 4	0.0, 0.2, 1.2	NA	NA
	40–60	0, 0, 1	0.0, 0.0, 1.2	NA	NA
	60–80	0	0.0	0	0
	>80	0	0.0	0	0

Lower, median, and upper bound for the area exposure (in thousand km^2^), total population (in millions), and properties (in millions) on the US east coast. The rows without bounds are where the lower, median, and upper bounds have the same exposure. The population and property exposure are only estimated for counties with an area exposure >60%. NA (not applicable) is assigned as the population and property exposure for counties with an area exposure of 60% or less. The area exposure, total population, and properties for each county are summarized in Tables S2–S5.

Our analysis shows that out of the 172 counties analyzed on the US east coast, 154 to 163 counties are exposed to subsidence rates >0 mm per year, 138 to 163 counties to rates >1 mm per year, and 56 to 152 counties to rates >2 mm per year (considering the lower to upper bounds) (Figs. 3, S6, and S7A–C). However, only 6 to 32 counties have a high percent area (>60%) exposed to land subsidence for the different subsidence hazard severity (subsidence rates >0 mm per year to >2 mm per year). These counties include some major metropolitan areas on the US east coast, including Hampton (VA), Norfolk (VA), Virginia Beach (VA), Chesapeake (VA), Baltimore City, (MD), Union (NJ), Middlesex (NJ), Monmouth (NJ), Ocean (NJ), New Haven (CT), and several counties in New York City (Queens, Bronx, Nassau, and Westchester; Figs. 3A–C and S6–S8). These communities with a high percent area exposed to subsidence also have high estimated population and property exposure, with 242,000 people and 95,000 properties for Norfolk (VA), 451,000 people and 177,000 properties for Virginia Beach (VA), 826,000 people and 335,000 properties for Baltimore city (MD), 822,000 people and 294,000 properties for Middlesex (NJ), and ∼5 million people and ∼1.8 million properties for Queens, Bronx, and Nassau (NY) (Table S2). We estimate that a total land area between 2,200 and 81,000 km^2^, a population between 1.2 and 16 million people, and 476,000 and 6.3 million properties on the US east coast's coastal communities are exposed to subsidence rates of 0 to 2 mm per year (Table 1). However, as the severity of subsidence hazard increases, the extent of exposure decreases. For exceptionally high subsidence rates >3 mm per year, we find a total exposed land area between 74 and 14,400 km^2^. However, only one county—Sussex, DE (upper bound)—exhibits a high percentage of area exposure (>60%; Table 1 and Fig. S7D).

### Infrastructure subsidence exposure analysis

Detecting and monitoring critical infrastructure affected by fast subsidence rates are essential in ensuring their structural integrity, maintaining their longevity, and monitoring site-specific structures vulnerable to land subsidence and related hazards (28, 32). On the US east coast, we analyzed the exposure to subsidence for 14 infrastructure systems, including transport networks (roads and railways), flood-control systems (levees and dams), health facilities (hospitals and nursing homes), and other critical infrastructure (airports, schools, universities, train stations, fire departments, police stations, post offices, and libraries; see Materials and methods). Our analysis of subsidence exposure for infrastructure is dependent on the infrastructure feature type, i.e. linear (e.g. roads), point (e.g. dams), or polygon (e.g. hospitals; see Materials and methods).

**Fig. 4.**
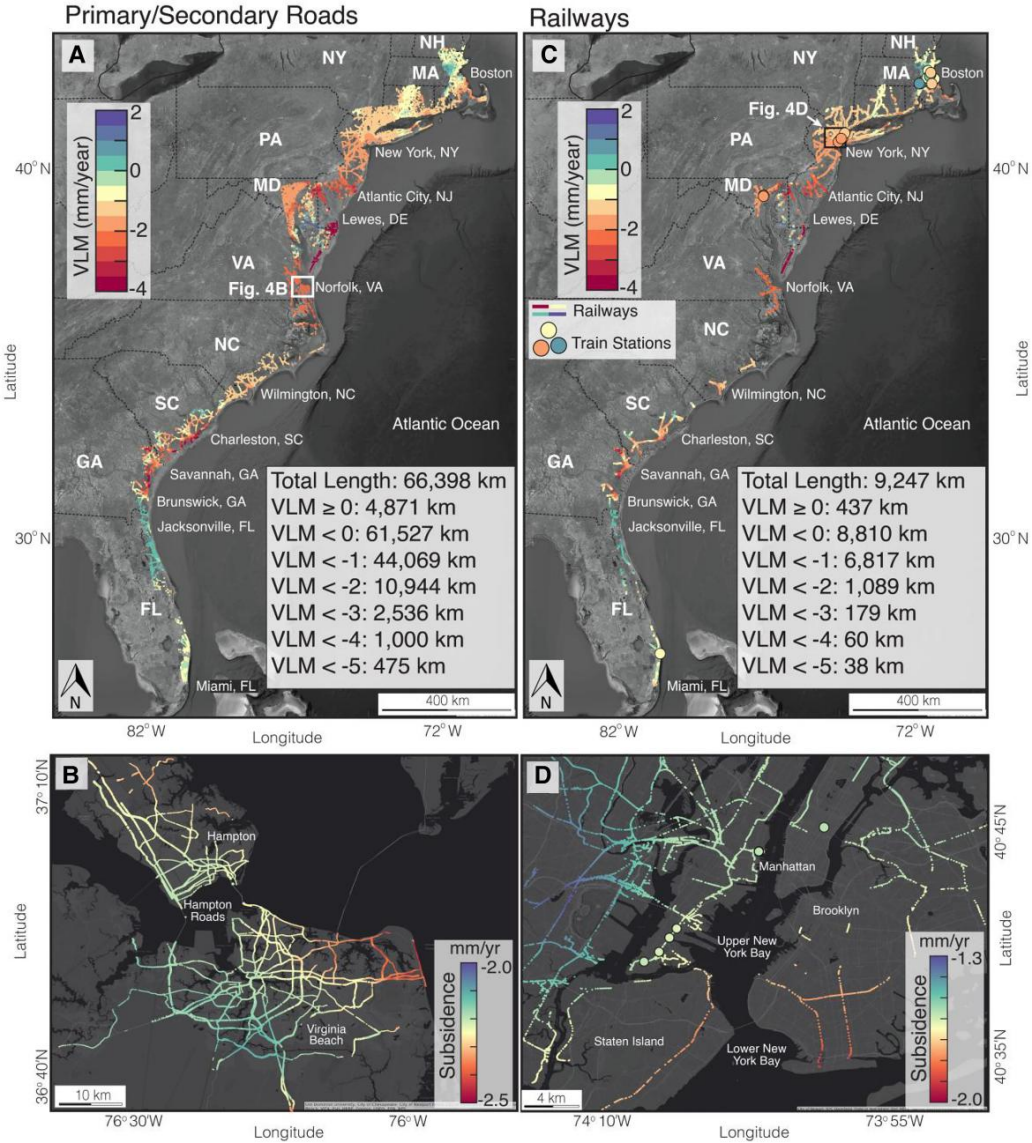
Subsidence exposure for roads and railways on the US east coast. A) Median exposure to subsidence for primary/secondary roads. Exposure analysis for primary and secondary roads is summarized in (A). The lower and upper bounds for the primary and secondary roads exposure analysis are shown in Fig. S9. White rectangle indicates the extent of (B). B) Primary and secondary roads affected by subsidence in Hampton and Norfolk (VA). C) Median exposure to subsidence for railways and train stations. Exposure analysis for railways is summarized in (C). The lower and upper bounds for the railways exposure analysis are shown in Fig. S10. Black rectangle indicates the extent of (D). D) Railways are affected by subsidence in New York City (NY). The circles in (C and D) are the locations of train stations. Background Images in (A) and (C) are from Google, Earthstar. Background Images in (B) and (D) are from ESRI, HERE.

**Fig. 5.**
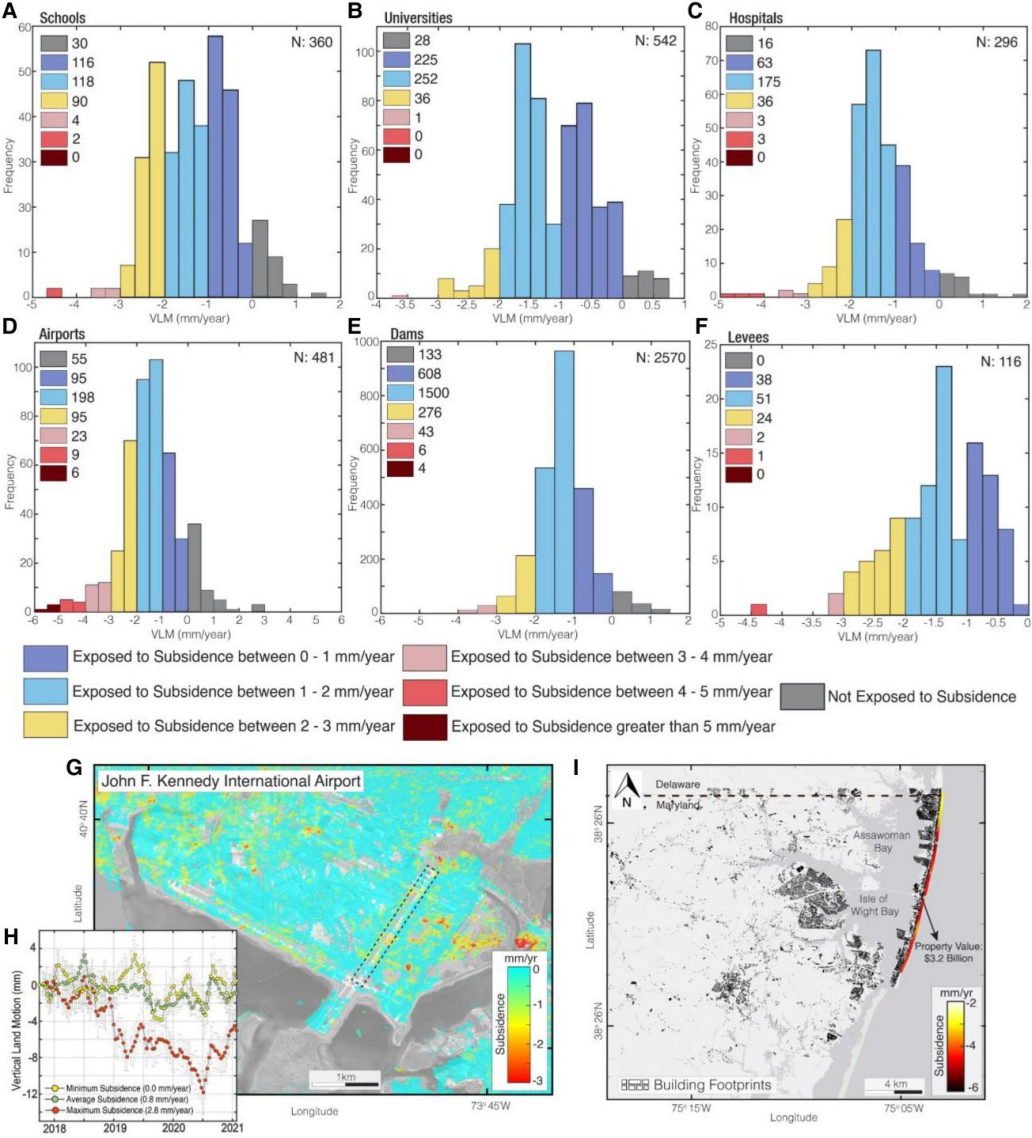
Subsidence exposure for infrastructure facilities on the US east coast. Histogram showing average weighted subsidence exposure for A) schools, B) universities, C) hospitals, D) Airports, E) dams, and F) levees. The number of infrastructure facility within each colored histogram is shown in the histograms. Not exposed to subsidence means VLM > 0 mm per year. *N* is the number of infrastructure facilities. G) Areas affected by subsidence in John F. Kennedy International airport. H) Time series of vertical land motion (VLM) around the runway highlighted as the dotted black rectangle in (G). I) Subsidence across the Atlantic coast Maryland shoreline levee system. The black polygons are building footprints. The property value of buildings behind the levee is US$3.2 billion. Background Images in (I) is from ESRI, HERE.

Subsidence exposure analysis of infrastructure assets for 14 states along the US east coast is shown in Figs. 4, 5, and S9–S11. Figures 4A and S9 show the distribution of vertical deformation velocities over the primary and secondary roads. The exposure analysis shows that between 77 and 99% (5,300 to 6,900 km out of 6,942 km) of interstate highways and 76 and 99% (50,000 to 65,000 km out of 66,000 km) of primary and secondary roads are exposed to subsidence (VLM < 0 mm per year) on the US east coast. High subsidence rates (>3 mm per year) are observed on 2.3 to 444 km of interstate highways and 490 to 8,500 km of primary and secondary roads on the US east coast, observed particularly along the coastal fringes of Georgia (GA), SC, NC, DE, and the Delmarva Peninsula (Figs. 4A and S9). Figure 4B highlights a critical section of roads in Hampton and Norfolk (VA), where subsidence rates along the roads exceed 2 mm per year. Roads in the Hampton area have documented increased flooding of transport infrastructure due to progressive subsidence and SLR in the region (45–47).

The railway network density on the US east coast is among the highest in the country, particularly in New England, which includes six Northeastern states: CT, Maine, MA, New Hampshire (NH), RI, and Vermont. The exposure analysis for railway systems and train stations is shown in Figs. 4C and S10. The exposure analysis shows sinking on 81 to 99% of the railway systems (7,452 to 9,221 km out of 9,247 km) and 42% (11 out of 26) of train stations (Fig. S11B), with subsidence rates of >3 mm per year observed along 41 to 846 km stretch of railways on the US east coast. The high density of underground railways (subways) in New York City increases the risk associated with the observed subsidence hazard in this region (Fig. 4D).

Figures 5 and S11 show the subsidence exposure analysis for an ensemble of infrastructure facilities (schools, universities, hospitals, airports, dams, levees, libraries, post offices, fire departments, police stations, and nursing homes) on the US east coast. In Figs. 5(A–F) and S11, the warming of the colors from blue to red denotes an increased subsidence hazard severity. Subsidence exposure analysis shows that >70% of infrastructure in each category is sinking. However, we find that <10% of the infrastructure facilities on the US east coast are exposed to subsidence >3 mm per year. Subsidence hazards can be a major concern for specific high-risk infrastructures, such as dams, levees, or airports.

Table 2 summarizes the statistics for subsidence exposure for 10 levees protecting some of the most valuable properties on the US east coast. Median subsidence rates >1 mm per year affect all the levee systems, with a total estimated at-risk population of ∼46,000, ∼27,000 buildings, and properties valued at US$12 billion. Note that this population and property exposure are only the base value estimated from the 10 highlighted levees (Table 2). The actual population and property exposure from the 116 levees exposed to subsidence (Fig. 5F) may be two to three times greater.

**Table 2.** Subsidence exposure for selected levees on the US east coast.

Name	Year of construction	Population exposure	Building exposure	Property value (US$ million)	Maximum subsidence rate (mm/year)	Median subsidence rate (mm/year)	Maximum angular distortion category
S-97 North Tie Back, FL	1964	2,248	1,253	272	1.14	1.02	Low
Alligator River Levee Ring, NC	NA	239	154	64.3	2.05	1.56	Low
Norfolk, Virginia Central, VA	1971	4,502	219	652	2.20	2.02	Low
Virginia Beach, VA	2000	1,991	254	241	2.46	2.43	Low
Atlantic Coast Maryland Shoreline, MD	1992	5,187	1,475	3,200	5.06	4.27	Low
Rahway, Rahway River South Branch RB, NJ	1966	1,447	430	459	1.61	1.60	Low
Raritan Bay and Sandy Hook Bay, Keansburg, NJ	1978	17,023	6,099	3,330	1.86	1.85	Low
Oakwood Creek West Bank, NY	2000	292	102	39.3	1.76	1.76	Low
Stamford HSPP, CT	1969	8,381	1,300	1,140	1.31	1.30	Low
New Bedford HSPP, MA	1966	4,624	16,359	2,690	1.54	1.47	Low

NA indicates not applicable. The data are not available from the national levee database provided by the USACE. The categories for the angular distortion (*β*) values include low (β<1/3,000), medium (1/3,000≤β<1/1,500), high (1/1,500≤β≤1/500), and very high (β>1/500). Note that the national levee database only includes a small percentage of existing levee systems (48).

Major airports on the US east coast exposed to subsidence are John F. Kennedy International Airport (JFK), with a median subsidence rate of 1.7 mm per year, LaGuardia Airport with a median subsidence rate of 1.5 mm per year, Newark Liberty International Airport (EWR) with a median subsidence rate of 1.4 mm per year, and Boston Logan International Airport with a median subsidence rate of 1.1 mm per year. To emphasize the potentially damaging effect of subsidence on airports and levees, we highlight the subsidence on JFK airport and the Atlantic coast Maryland shoreline levee (Fig. 5G–I). JFK airport, which serves >60 million annual passengers, shows a subsidence rate >2 mm per year in several areas of the airport and average and maximum subsidence rates of 0.8 and 2.8 mm per year, respectively, on the runway (Fig. 5G and H). The Atlantic coast Maryland shoreline levee protects some of the most valuable assets (US$3.2 billion) on the US east coast and also has the greatest subsidence exposure for levees, with a maximum subsidence rate of 5.06 mm per year and a median subsidence rate of 4.27 mm per year (Fig. 5I and Table 2).

### Infrastructure differential subsidence hazard analysis

Analysis of the differential subsidence hazard for infrastructure on the US east coast revealed that most infrastructure systems are in low (β<1/3,000) hazard areas. Specifically, all railroads, interstate roads, levees, nursing facilities, train stations, fire departments, post offices, libraries, and police stations are located in areas with low values (Fig. S12). In the Delmarva peninsula, however, 14 km stretch of primary and secondary road with medium (1/3,000≤β<1/1,500) hazard levels were identified. Furthermore, infrastructure facilities with *β* values exceeding 1/3,000 (i.e. medium-to-high -hazard zones) include 12 school buildings, 4 university buildings, 1 hospital, 51 airports/airfields, and 117 dams (Fig. S12A–E). These structures are potential facilities for careful monitoring and management to assess potential structural damage due to subsidence gradients. Note that since the angular distortion (*β*) relies on variations between neighboring pixels, we are limited to the smallest detectable scale of ∼50 m (see Materials and methods). Assessing localized tilts and structural damage on specific infrastructure would require a finer spatial resolution (29).

## Discussion

This study assesses the impact of subsidence on communities on the US east coast. We comprehensively analyzed subsidence exposure on coastal infrastructure systems and county-specific population and property exposure on the US east coast. The VLM rate map highlights substantial subsidence (VLM < 0 mm per year) and exposure along the US east coast, affecting 89 to 95% of the counties (154 to 163 out of 172) analyzed in this study and a total land area between 59,000 and 81,000 km^2^ (Table 1). However, only 7 to 43 counties and a total land area between 74 and 3,700 km^2^ on the US east coast are exposed to high subsidence rates (VLM < −5 mm per year) (Table 1). This substantial decrease in the exposed areas is important and suggests that, while sinking land is widespread on the US east coast, the severity of exposure to land subsidence may not necessarily be pervasive. This raises the question: what annual subsidence rate threshold should be cause for concern to policymakers and citizens? Currently, there is no universally accepted threshold for the subsidence rate to define associated hazard severity and answers to this question would require a concerted effort from the global subsidence research community (e.g. the International Panel on Land Subsidence) (49). Exposure and the risk from land subsidence hazards depend on various factors, including the land subsidence duration and rate variations, present land elevation, type, use, tolerance levels of infrastructure, population/property density, and occurrence of compounding hazards. Generally, a subsidence rate of more than a few millimeters per year could cause concern, particularly in densely populated areas or areas with essential facilities like hospitals, schools, or transportation hubs.

However, it is important to note that exposure to subsidence does not necessarily imply structural damage. Differential subsidence, which results from the uneven settlement of the land, presents a greater hazard to infrastructure. It is less likely that infrastructure will be damaged if the rate of land subsidence is uniform across an area (43). On the other hand, the risk of structural damage increases as the subsidence gradient increases (29, 43). Our analysis shows that on the US east coast, land is sinking evenly, with differential subsidence classified as low (*β* < 1/3,000) for >99.5% of the land area analyzed in this study. However, note that the calculated *β* reflects a partial distortion that occurred over the 13-year InSAR coverage period, not the total distortion. Unmitigated, such subsidence may slowly but surely compromise the structural integrity of urban infrastructure (29) and exacerbates other hazards (e.g. flooding), contributing to socioeconomic losses (3, 50).

The percent land area within each county affected by subsidence on the US east coast has important implications for flood frequency and severity in the different communities. Land subsidence can potentially increase the flooded area during coastal storm events by modifying the base flood elevations and topographic gradients (50). Major metropolitan communities with a high percentage area (>60%) exposed to subsidence rates >1 mm per year, such as Hampton (VA), Portsmouth (VA), Norfolk (VA), Baltimore City (MD), Newark (NJ), and New York City (NY) (Fig. 3B) are affected yearly by persistent nuisance flooding events (17, 45, 47, 51–55) (Fig. S13 and Table S2) and nuisance flooding will increase dramatically (∼3- to 12-fold) by 2050 (7). Such flooding disrupts economic activities, resulting in fiscal losses worth billions of dollars from damaged properties, flood insurance payout, and the loss of lives. In these communities, the presence of infrastructure may exacerbate the loss associated with land subsidence-related hazards.

Infrastructure damage due to subsidence has direct, indirect, short-term, and long-term consequences, such as disruptions of clean water supplies, transportation, education, and health care, economic stagnation, and a severe death toll (47). The hazards associated with the subsidence exposure observed in our analysis are heightened by the potential consequences. For instance, New York City's railway systems show subsidence >1.5 mm per year (Fig. 4D). Since railways in New York City are predominantly underground, potential inundation from relative SLR due to subsidence may cause the irreversible loss of this infrastructure in the future. The US east coast is densely fortified with flood-control dams and levees. An assessment of available meta-data for the dams analyzed in this study shows that 1,756 out of 2,570 (68% of dams) are classified as high/significant hazard potential to downstream areas (Fig. S14). This hazard potential is a measure of the probable loss of life and the economic and environmental consequences of infrastructure failure. Likewise, an assessment of 10 levees across 8 states, which protects some of the most valuable properties on the US east coast, shows maximum subsidence rates on the levees between 1.14 and 5.06 mm per year (Table 2). Continuous subsidence combined with accelerating SLR indicates a future of continuous maintenance and enhancements for levees, seawalls, and dams to offset elevation loss and retain their structural integrity and utility (16).

In absolute terms, subsidence is a major threat worldwide and rapid urbanization of coastal zones leads to increased exposure of coastal communities and infrastructure (10, 14, 15, 33). Beyond the direct impacts, subsidence in coastal zones is a major driver of current and future hazards, amplifying the impacts of climate change-driven global SLR (3, 16, 17, 56). Consequently, understanding the impact of continuous subsidence in coastal zones is essential in transitioning from reactive to proactive climate change mitigation strategies. For a proactive approach to be effective, critical indicators of change, such as subsidence, must be monitored regularly (57). This monitoring and management are crucial to avoiding a colossal future “climate change tax.” The cost of an ineffective climate policy is reflected in the economic consequences of climate tipping points. The concept of tipping points in a changing climate is relevant in addressing the critical threshold at which a tiny perturbation can alter the state of a system (58). The potential economic implications of such tipping points are significant, with economic losses in trillions of dollars (59, 60). So far, these tipping elements in earth systems are only examined within the context of climate systems (58, 61) and ecosystems (62–66), with no considerations for the lithospheric component. We introduce coastal land elevation as the lithospheric component of tipping elements in the earth systems. Considerations for the loss of coastal elevation are crucial in accounting for future economic losses. Even if current climate measures successfully curb rising sea levels, continuous land subsidence may result in irreversible inundation and more frequent flooding in some coastal regions (17). Such coastal land elevation loss with current sea levels is sufficient to trigger annual rates of exceedances over elevation thresholds from mean higher high water (67), resulting in increased frequency of flooding and higher nuisance flood levels, overwhelming local and federal resources, devastating communities, and increasing climate economic losses in coastal cities. Within the context of sustainable subsidence governance, disentangling total subsidence from anthropogenic subsidence is critical to proactive decision-making. If subsidence is solely the result of nonanthropogenic processes (e.g. GIA), then subsidence mitigation policies would be largely ineffectual. The observed subsidence on the US east coast is attributable to ongoing GIA and non-GIA processes (18). Thus, subsidence governance is a crucial sustainable mitigation strategy for minimizing the exposure of coastal communities on the US east coast.

Our analysis demonstrates the potential role of remotely sensed observations in asset management. Unlike traditional field surveys and on-site instrumentation techniques, monitoring infrastructure network systems through remote sensing technology offers a superior advantage in large-scale, high-resolution, and increased frequency monitoring. This dataset and other high-resolution remotely sensed datasets (e.g. the soon-to-launch NISAR satellite) can augment current visual inspection standards for planning, operations, maintenance, and development of infrastructure programs. In addition, the use of remote sensing is critical for deficiency identification, damage preventative risk assessment, and post-hazard response because it provides up-to-date infrastructure information to monitor the state of constructed infrastructure. Recently, a significant infrastructure bill was signed into law in the United States, which includes investment to fund the repairs of roads and bridges and support other major transformational projects. Our results and similar spatiotemporal remotely sensed observations can provide quantitative data to guide the disbursement of the investment.

## Materials and methods

### VLM data

To create a map of VLM rates whose uncertainties are verified and qualities are assessed, we leverage the ∼50 m resolution VLM datasets from 2007 to 2020 published by the USGS (39, 40) and Ohenhen et al. (41) (Fig. S1A). The VLM datasets were produced using an interferometric dataset comprising 3,057 SAR images from ascending tracks of the Sentinel-1 A/B and ALOS-1 satellites, in combination with observations of 3D displacement fields at 162 GNSS stations (Fig. S2). To produce the spatially continuous VLM maps across the region, two large-scale maps of line-of-sight (LOS) velocity for Sentinel-1 A/B and ALOS-1 satellites were first produced. The LOS displacements were generated for 17 Sentinel-1 and 95 ALOS-1 frames using a multitemporal Wavelet-Based InSAR (WabInSAR) algorithm (68–71). To this end, the wavelet-based analysis applied to the ∼20,000 interferograms involved identifying and removing noisy pixels, reducing the effects of topographically correlated atmospheric phase delay, and spatially uncorrelated digital elevation model (DEM) error (68, 69). Next, the LOS velocity for each pixel was estimated as the slope of the best fitting line to the associated time series using a reweighted least-squares estimation. Lastly, the LOS velocity for each frame is mosaiced to generate large-scale continuous LOS displacements for Sentinel-1 and ALOS datasets following the procedure of Ohja et al. (72). Further details on the model input parameters, interferogram, and LOS velocity generation can be found in Ohenhen et al. (41).

To combine the LOS velocities with the GNSS 3D displacements and generate a map of the VLM rate and assess uncertainties, we adopted a variation of the stochastic model proposed by Ohenhen et al. (41):


(1) $$\left(\begin{array}{c} y_{a}\\ y_{s}\\ \begin{array}{c} E_{G}\\ \begin{array}{c} N_{G}\\ U_{G} \end{array} \end{array} \end{array}\right)=\left(\begin{array}{ccc} C_{a}^{E} & C_{a}^{N} & C_{a}^{U}\\ C_{s}^{E} & C_{s}^{N} & C_{S}^{U}\\ \begin{array}{c} 1\\ 0\\ 0 \end{array} & \begin{array}{c} 0\\ 1\\ 0 \end{array} & \begin{array}{c} 0\\ 0\\ 1 \end{array} \end{array}\right)\left(\begin{array}{c} E\\ N\\ U \end{array}\right),$$

where *C* represents the unit vectors projecting the 3D displacements onto the LOS (73), $E_{G}$, $N_{G}$, and $U_{G}$ are the interpolated GNSS velocities, $y_{a}$ and $y_{s}$ are the interpolated LOS velocities and $\sigma_{a}^{2}$ and $\sigma_{s}^{2}$ are the variances for each pixel, where the subscripts *a* and *s* indicate ALOS-1 and Sentinel-1, respectively, and *E*, *N*, *U* are unknowns. Using matrix notations,


(2) $$X=(E\;N\;U{)}^{T}$$

(3) $$=\left(\begin{array}{ccc} C_{a}^{E} & C_{a}^{N} & C_{a}^{U}\\ C_{s}^{E} & C_{s}^{N} & C_{S}^{U}\\ \begin{array}{c} 1\\ 0\\ 0 \end{array} & \begin{array}{c} 0\\ 1\\ 0 \end{array} & \begin{array}{c} 0\\ 0\\ 1 \end{array} \end{array}\right)$$

(4) $$L={\left(\begin{array}{ccc} y_{a} & y_{s} & \begin{array}{ccc} E_{G} & N_{G} & U_{G} \end{array} \end{array}\right)}^{T}$$

(5) $$P=\left(\begin{array}{ccc} \frac{1}{{\left(\sigma_{a}\right)}^{2}} & 0 & \begin{array}{ccc} 0 & 0 & 0 \end{array}\\ 0 & \frac{1}{{\left(\sigma_{s}\right)}^{2}} & \begin{array}{ccc} 0 & 0 & 0 \end{array}\\ \begin{array}{c} 0\\ 0\\ 0 \end{array} & \begin{array}{c} 0\\ 0\\ 0 \end{array} & \begin{array}{c} \begin{array}{ccc} \frac{1}{{\left(\sigma_{E}\right)}^{2}} & 0 & 0 \end{array}\\ \begin{array}{ccc} 0 & \frac{1}{{\left(\sigma_{N}\right)}^{2}} & 0 \end{array}\\ \begin{array}{ccc} 0 & 0 & \frac{1}{{\left(\sigma_{U}\right)}^{2}} \end{array} \end{array} \end{array}\right).$$

The optimized value of unknown in terms of least-squares and associated variance–covariance matrix ($Q_{XX}$) are given by


(6) $$X=(A^{T}PA{)}^{-1}A^{T}PL,$$

(7) $$Q_{XX}=\frac{r^{T}Pr}{df}(A^{T}PA{)}^{-1},$$

where *A* is Green’s function, *L* is the observations, and *P* is the weight matrix, which is inversely proportional to the observant variance ($\sigma^{2}$), *df* is the degree of freedom, which is equal to the number of independent equations minus the number of unknowns and r=L−AX. The weight matrix, *P* assigned to the interpolated GNSS observation, is a weight proportional to the nearest GNSS station (41).

Next, we perform a χ^2^ goodness of fit test to examine whether the unknown parameters are distributed normally, or their distribution is skewed due to outliers and systematic errors (74). This test accounts for the impact of observations error and model inaccuracy on the quality of the estimated VLM rates used in the exposure analysis. To that end, we implemented a statistical hypothesis testing with a null hypothesis stating $r^{T}Pr$ follows a χ^2^ distribution ($\chi^{2}$) with *df* degree of freedom (75). Thus, the alternative hypothesis is defined as


(8) $$r^{T}Pr>\chi_{df}^{2}.$$

We attempt to reject the null hypothesis at a significance level of *α* = 0.05, namely, we excluded pixels where $r^{T}Pr$ is greater than the critical value (Fig. S1B). This exclusion criterion was applied to ensure the robustness of our exposure analysis by removing data points that may be affected by measurement errors or model deficiencies. Generally, we note higher χ^2^ values in areas with noted higher standard deviations. This could be attributed to several factors: the nonlinearities in surface deformation rates observed between the ALOS-1 and Sentinel-1 periods, the sparse distribution of GNSS stations, and the comparatively higher standard deviation observed at these GNSS stations in these areas (76). The final VLM utilized in this study is shown in Fig. 2A. The distribution of the standard deviation shows that 79% of the pixels have values <1 mm per year, and >99% of the pixels have standard deviation values <3 mm per year (Fig. S4A, B). InSAR VLM validation was performed using 30 independent GNSS stations provided by the Nevada Geodetic Laboratory (77) (Fig. S2). The comparison against the GNSS vertical observation shows a mean and a standard deviation of −0.15 mm per year and 0.8 mm per year, respectively, for the difference between the two datasets (Fig. S4C).

### SAR analysis of JFK airport

To measure the high-resolution surface deformation at JFK airport, we analyzed 104 SAR images acquired during 2016 May 17 to 2021 February 8 in Ascending orbit of the Sentinel-1A/B satellite. We applied a multilooking factor of 6 × 1 in range and azimuth directions, resulting in a ground resolution cell of ∼15 m. The rest of the analysis is similar to that described above for mapping large-scale VLM rates along the US east coast.

### Differential subsidence data for the US east coast

We define hazard due to differential subsidence on the US east coast using angular distortion (*β*), which is a measure of differential settlement between adjacent pixels (27, 78). Given *l*, the distance between adjacent SAR pixels and Δδ as the differential settlement between *l* and *β* is defined as the ratio of Δδ to *l*:


(9) $$\beta =\frac{\Delta \delta \;}{l},$$

where *l* is 50 m and Δδ is computed for the InSAR period, 2007–2020 (13 years). The obtained *β* values are dimensionless and expressed in fractions. Following Cigna and Tapete (29), we define four categories indicative of the increasing hazard severity, viz: low (β<1/3,000), medium (1/3,000≤β<1/1,500), high (1/1,500≤β≤1/500), and very high (β>1/500). High *β* values identify areas of high subsidence gradients between adjacent points and indicate a higher likelihood of damage (29, 43, 44).

### Population, properties, and infrastructure data

The population and properties dataset for the US east coast is based on the open-access TIGER/Line demographic and economic data record available from the US Census Bureau. For the population data, we extracted the 2021 population estimates available on a by-county basis for 172 counties and 14 states along the US east coast, including FL, GA, SC, NC, VA, MD, DE, Pennsylvania, NJ, NY, CT, RI, MA, and NH. A summary of the 172 selected counties is shown in Tables S2–S5. The properties dataset is from the 2020 total housing units categorized by the census for each of the 172 counties.

We analyzed the exposure to subsidence for 14 infrastructure systems on the US east coast, including transport networks (roads and railways), flood-control systems (levees and dams), health facilities (hospitals and nursing homes), and other critical infrastructure (airports, schools, universities, train stations, fire departments, police stations, post offices, and libraries). The infrastructure dataset used in this study is from three sources. All roads (primary/secondary and interstate highways), railways, train stations, airports/airfields, universities, schools, hospitals, nursing homes, fire departments, post offices, libraries, and police stations data are based on open access data from TIGER/Line Shapefiles available from the US census bureau. The dams are extracted from the United States Army Corps of Engineers (USACE) National Inventory of dams, which contains an extensive dataset of 92,092 dams across the United States. The levees dataset is from the USACE national levee database, which contains a total of 6,972 levee systems (length: 39,445 km) across the United States. However, we note that the USACE national levee database only includes a small percentage (∼20%) of existing levee systems in the United States (48). The roads, railways, and levees datasets are linear/line features. The datasets of the dams, train stations, airports/airfields, schools, fire departments, post offices, libraries, and police stations are point features. While the universities, hospitals, and nursing homes are area/polygon features. Figure 1 provides summary statistics of all infrastructure data used in this study.

### Subsidence exposure analysis

Exposure indicates the degree to which the elements-at-risk (population, properties, and infrastructure) are exposed to a particular hazard (79). In this study, we define exposure to land subsidence based on the magnitude of negative land level change (VLM < 0 mm per year) for the different elements-at-risk. We present the subsidence exposure for several subsidence rates in order of increasing hazard severity: VLM < 0 mm per year, VLM < −1 mm per year, VLM < −2 mm per year, VLM < −3 mm per year, VLM < −4 mm per year), and VLM < −5 mm per year.

To determine the area exposure to subsidence, we estimated the areas affected by subsidence hazard for 172 counties with InSAR pixels along the US east coast. To more precisely define the exposure limits in each county/infrastructure, we incorporated the standard deviation values associated with the VLM data (i.e. ±standard deviation). This approach yields estimates of the lower (VLM + standard deviation), median (VLM), and upper (VLM—standard deviation) bounds of the exposure analysis, which is critical for accurately assessing the impact across varying degrees of subsidence severity. We evaluated the exposure to subsidence for each county by implementing a regular grid of 100 m for each county and assigned the VLM rate for each grid cell as the median VLM value of InSAR pixels within that grid cell. Assuming spatially continuous subsidence and applying a linear interpolation, we then calculate the area exposed to subsidence for different subsidence hazard severity (i.e. VLM < 0 mm per year, VLM < −1 mm per year, VLM < −2 mm per year, VLM < −3 mm per year, VLM < −4 mm per year, and VLM < −5 mm per year) expressed as a percentage of the total area of the county (equation 8). Counties not exposed to subsidence for the different subsidence rate thresholds (i.e. VLM ≥ 0 mm per year, VLM ≥ −1 mm per year, VLM ≥ −2 mm per year, VLM ≥ −3 mm per year, VLM ≥ −4 mm per year, and VLM ≥ −5 mm per year) or without any InSAR pixels are given an absolute value of 0.


(10) $$\text{\%}\mathrm{Area}\;\mathrm{exposed}\;\mathrm{to}\;\mathrm{subsidence}=\frac{\mathrm{County}\;\mathrm{area}\;\mathrm{exposed}\;\mathrm{to}\;\mathrm{subsidence}}{\mathrm{Total}\;\mathrm{county}\;\mathrm{area}}\times 100\text{\%}.$$

Subsidence exposure for the infrastructure is independent of the infrastructure type, age, quality, tolerance, and other factors which may increase the risk. Exposure to subsidence for infrastructure is calculated based on the feature type (linear, point, or area). For the linear features (roads, railways, and levees), we generated a regular grid of VLM rates across the US east coast and extracted the VLM rate (and VLM ± standard deviation) of each point on the linear infrastructure. For the point features (train stations, airports/airfields, schools, fire departments, post offices, libraries, and police stations), we selected InSAR pixels within a radius of 500 m and calculated the average weighted VLM rate for each point infrastructure. Similarly, for the polygon features (universities, hospitals, and nursing homes), the VLM rate for each polygon infrastructure is the average weighted VLM rate of InSAR pixels within each polygon feature.

### Differential subsidence hazard analysis

The differential subsidence analysis for the linear (railways, roads, and levees) and area (universities, hospitals, and nursing homes) infrastructure was determined similarly to the exposure analysis described above. For the linear feature, we generated a regular grid of *β* values across the US east coast and extracted the *β* of each point on the linear feature. For each levee, we only report the maximum *β* category (low, medium, high, and very high). For the area infrastructure (universities, hospitals, and nursing homes), the *β* value for each area infrastructure is the maximum *β* of InSAR pixels within each polygon. For the point infrastructure, we extracted the *β* value in the grid corresponding to the location of the point feature.

## Supplementary Material

pgad426_Supplementary_Data

## Data Availability

The vertical land motion (VLM) rate data is made accessible through the Virginia Tech Data Repository at https://doi.org/10.7294/19350959. The angular distortion data is made accessible through the Virginia Tech Data Repository at https://doi.org/10.7294/22294672.v1. The population and properties dataset is available from the US census bureau (https://www.census.gov/geographies/mapping-files/time-series/geo/tiger-data.2010.html). The roads, railways, train stations, airports, universities, schools, hospitals, nursing homes, fire departments, post offices, libraries, and police stations data are available from the US census bureau (https://www.census.gov/cgi-bin/geo/shapefiles/index.php). The dam dataset is available from the United States Army Corps of Engineers (USACE) National inventory of dams (https://nid.sec.usace.army.mil/#/). The levee dataset is available from the United States Army Corps of Engineers (USACE) national levee database (https://levees.sec.usace.army.mil/#/). The flood events data is available from the Federal Emergency Management Agency (FEMA) flood risk database (https://www.fema.gov/data-visualization/historical-flood-risk-and-costs). All other data needed to evaluate the conclusions in the article are presented in the article and in supplementary materials.
