# Supplementary material for: Slowly but surely: Exposure of communities and infrastructure to subsidence on the US east coast
Source: PNAS Nexus. 2024 Jan 2;3(1):pgad426. doi: 10.1093/pnasnexus/pgad426 (PMC10759798; doi:10.1093/pnasnexus/pgad426)
Supplement: pgad426_Supplementary_Data [file pgad426_supplementary_data.pdf]

Supplementary Materials for

Slowly but Surely: Exposure of Communities and Infrastructure to Subsidence on the U.S. East Coast

Leonard Ohenhen *et al.*

*Corresponding author. Email: ohleonard@vt.edu

This PDF file includes:

Figs. S1 to S14
Tables S1 to S5

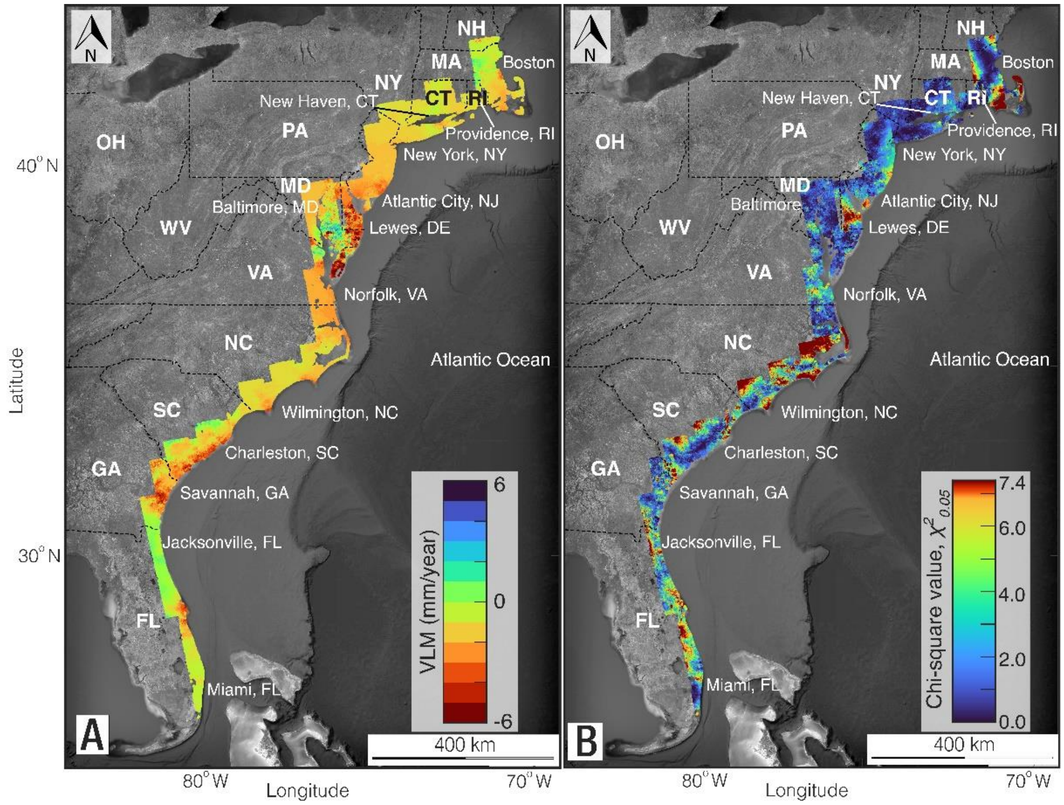

Fig. S1. Vertical Land Motion (VLM) along the U.S. east coast. (A) Estimated VLM rate from Ohenhen et al. (2023). This map includes ~38 million pixels at ~50 m resolution. **(B)** Chi-square values for hypothesis testing. Values are rejected when the Chi-square values greater than the critical value at a significance level of α = 0.05 (i.e. $\boldsymbol{\chi^2_{0.05}}$ = 7.4). The rejected values are the dark red colored pixels shown in supplementary Fig. 1B. The updated VLM rate for the U.S. east coast from this study is shown in Fig 2A. Background Images in (A) and (B) are from Google, Earthstar.

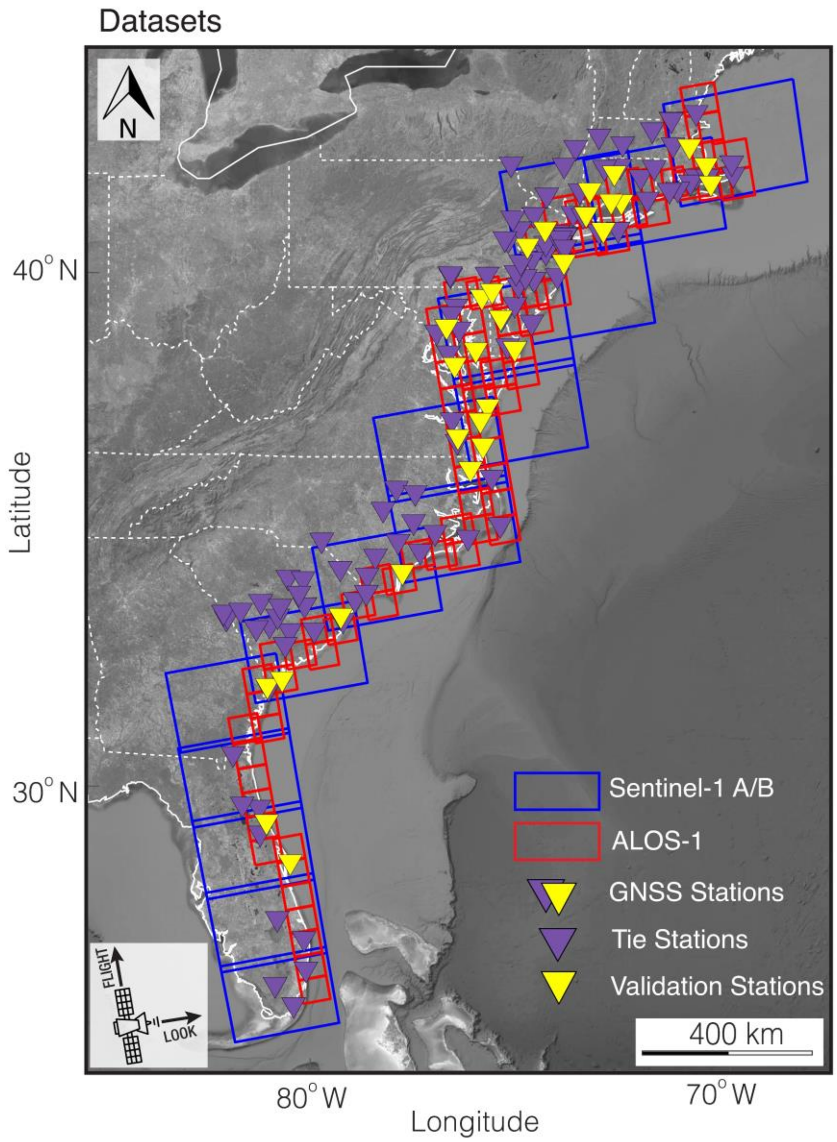

Fig. S2. Synthetic aperture radar (SAR) and global navigation satellite system (GNSS) datasets. The SAR dataset includes acquisition from Sentinel-1A/B C-band satellites (blue rectangles) spanning 2015 – 2020 obtained in ascending orbits and ALOS-1 L-band (red rectangles) spanning 2007– 2011 obtained in ascending orbits geometry. The GNSS dataset spans 2007 – 2020. The purple inverted triangles are the locations of GNSS tie stations used in the study. The yellow inverted triangles are the locations of 30 GNSS stations used in validation. Background Image is from Google, Earthstar.

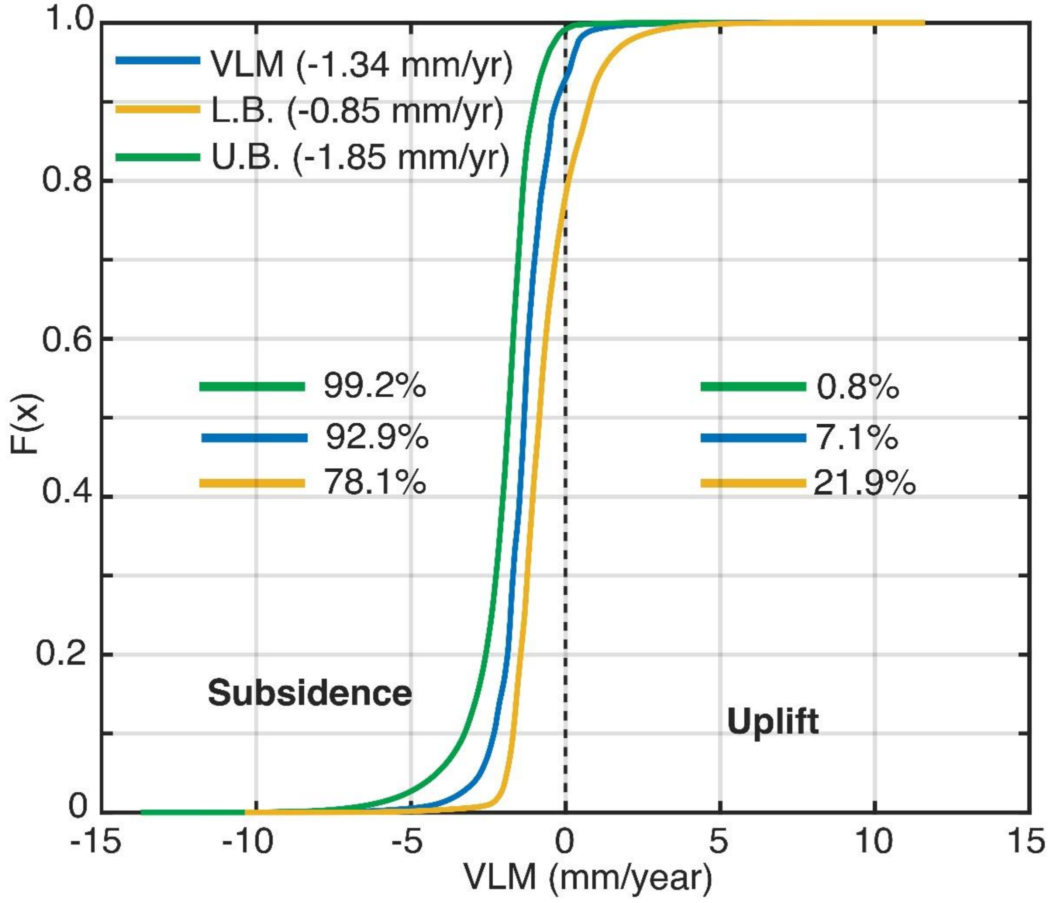

Fig. S3. Distribution of vertical land motion (VLM) rate. (A) Empirical cumulative distribution function for VLM, lower bound (L.B.) and upper bound (U.B.) (±1SD) of VLM evaluated using the standard deviation values shown Fig. S4A. The rates shown in parentheses are the median VLM rates.

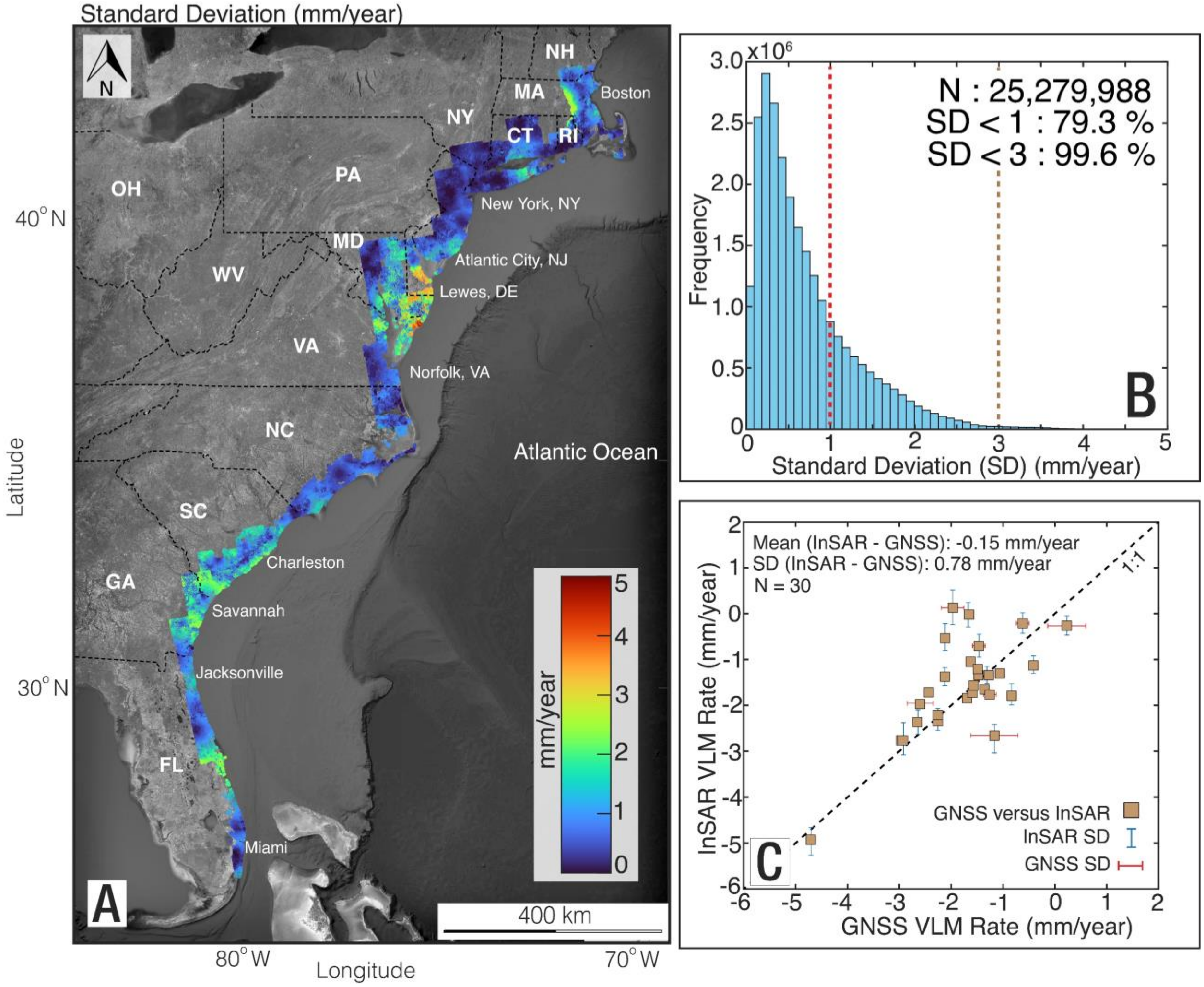

Fig. S4. Validation of vertical land motion (VLM). (A) VLM standard deviation (SD) distribution map. **(B)** Histogram showing the distribution of SD (mm per year). N is the number of pixels. The red and brown dotted lines show the location of SD = 1 mm per year and SD = 3 mm per year, respectively. **(C)** Bivariate plot comparing global navigation satellite system (GNSS) vertical rates with InSAR VLM rates. The comparison was obtained by averaging InSAR pixels within a radius of 100 m for each GNSS station. N is the number of GNSS validation station shown in Fig. S2. The SD of the difference between GNSS and InSAR dataset is 0.8 mm per year. Background Image in (A) is from Google, Earthstar.

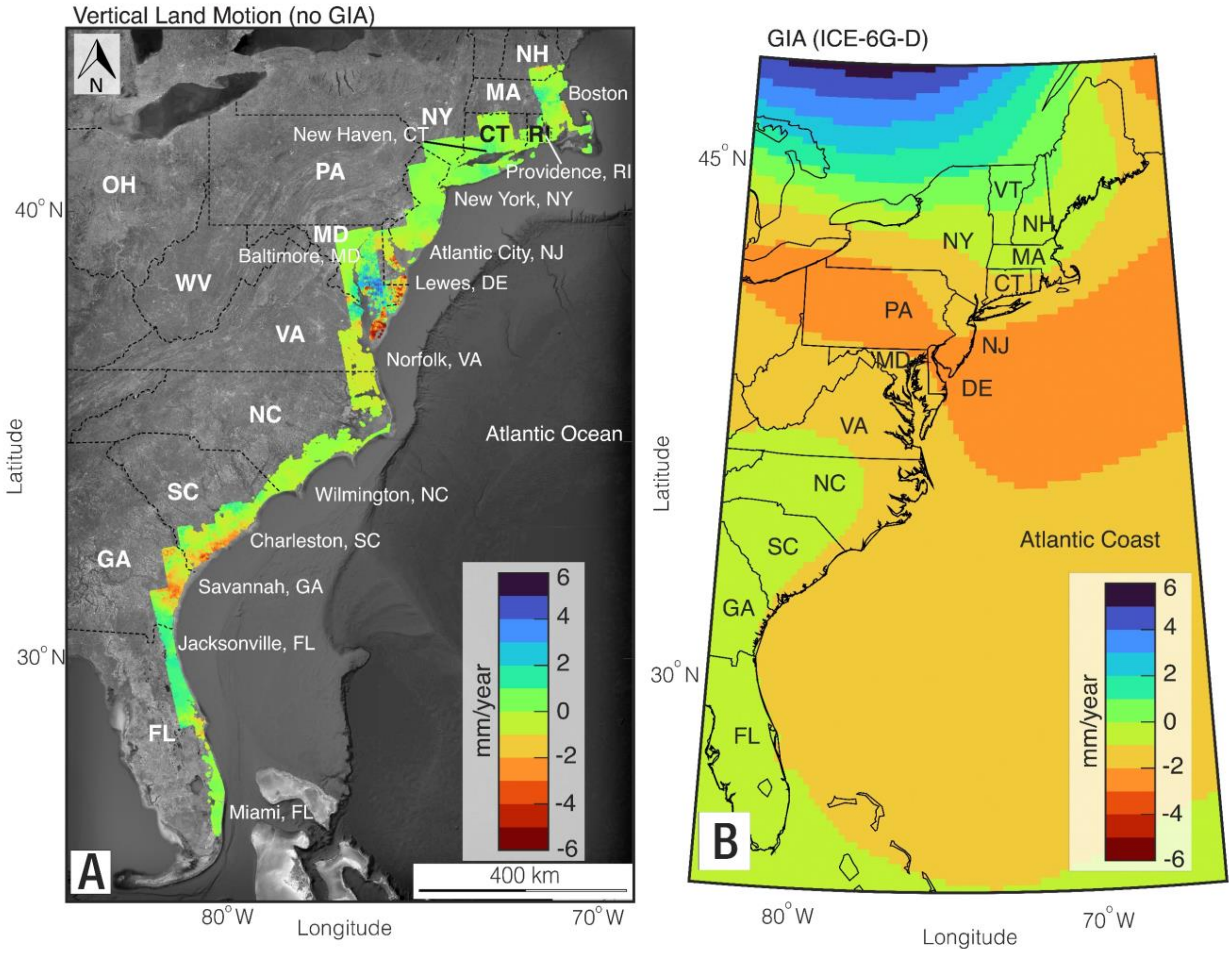

Fig. S5. Contribution of glacial isostatic adjustment (GIA) on vertical land motion (VLM). (A) VLM without the effects of GIA. Background Image is from Google, Earthstar. **(B)** GIA data from the ICE-6G-D model (*41*).

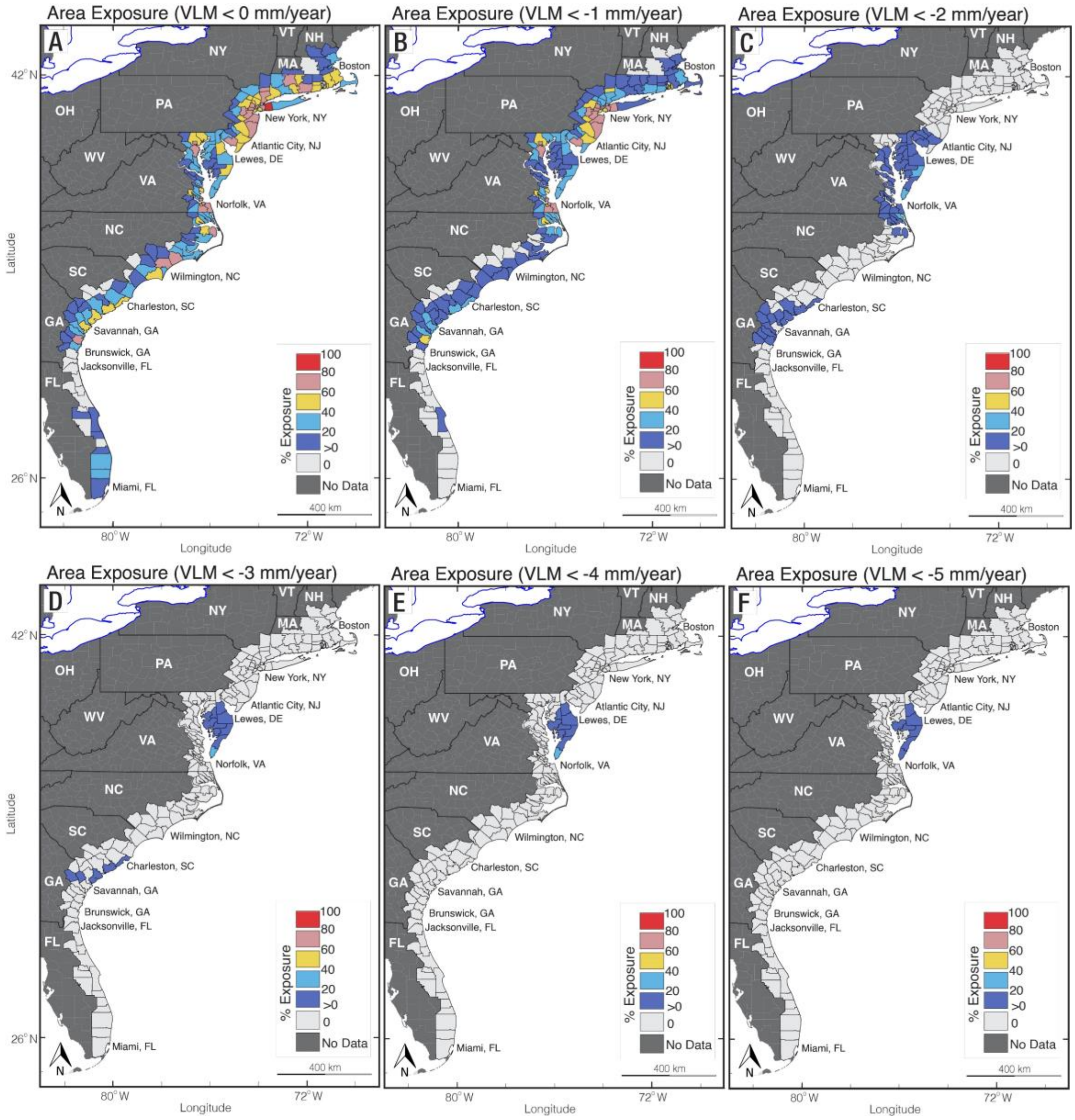

Fig. S6. Subsidence Area Exposure (lower bound) for Counties on the U.S. east coast. (A) Percentage of county's land area affected by VLM < 0 mm per year. **(B)** Percentage of county's land area affected by VLM < -1 mm per year. **(C)** Percentage of county's land area affected by VLM < -2 mm per year. **(D)** Percentage of county's land area affected by VLM < -3 mm per year. **(E)** Percentage of county's land area affected by VLM < -4 mm per year. **(F)** Percentage of county's land area affected by VLM < -5 mm per year. Note that this figure considers the lower bound (VLM + standard deviation), see Figs. 3 and S7 for the median (VLM) and upper bounds (VLM - standard deviation). The black and gray shape outline in Figs. 3A to F defines the extent of each county. The light grey counties in Figs. S6A to F show counties without any SAR pixels exposed to the different subsidence rates. Table 1 summarizes the number of counties, total land area in km^2, population, and properties exposed to subsidence. A summary table of all counties and land area exposure is provided in tables S3.

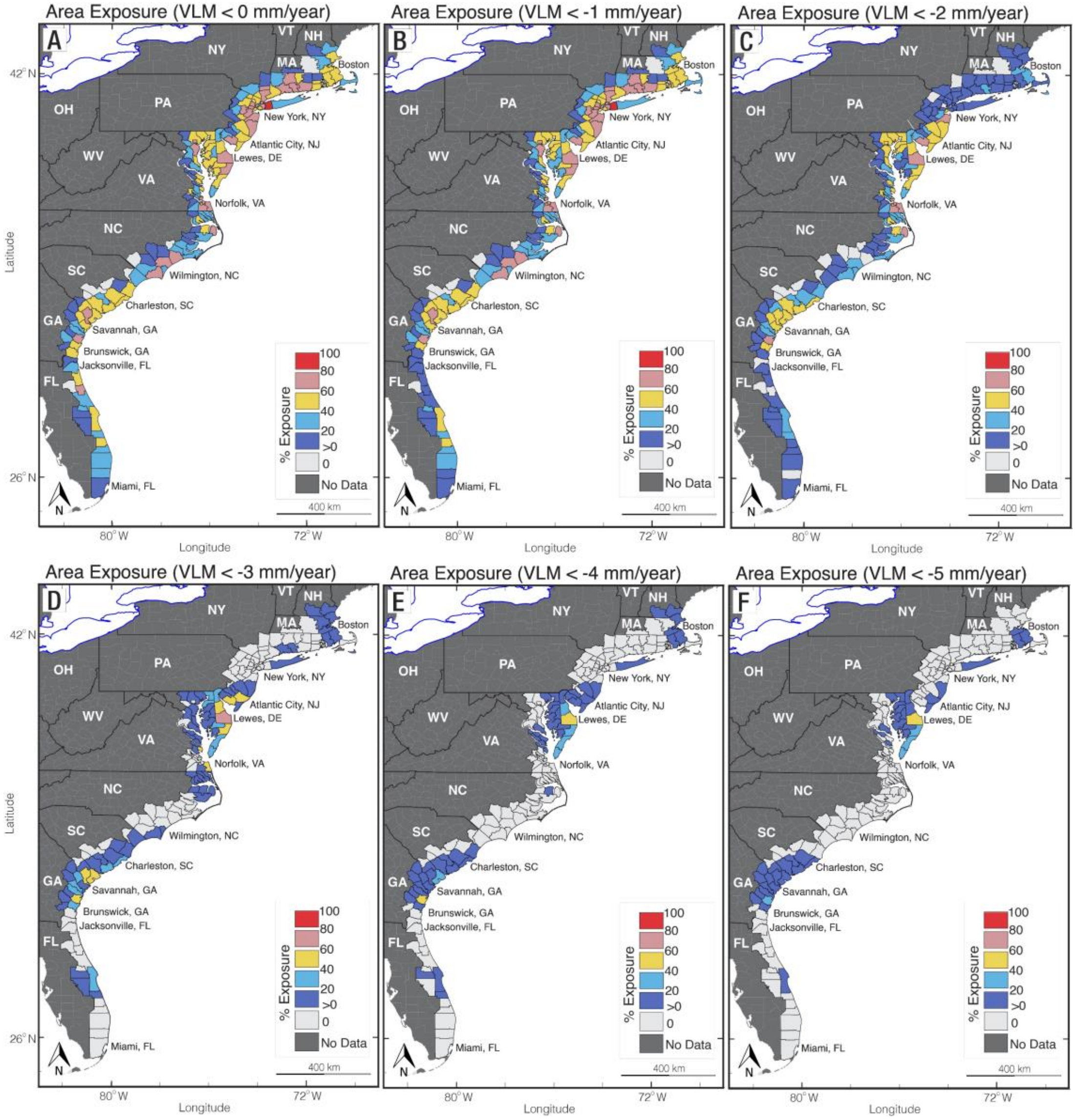

Fig. S7. Subsidence Area Exposure (upper bound) for Counties on the U.S. east coast. (A) Percentage of county's land area affected by VLM < 0 mm per year. **(B)** Percentage of county's land area affected by VLM < -1 mm per year. **(C)** Percentage of county's land area affected by VLM < -2 mm per year. **(D)** Percentage of county's land area affected by VLM < -3 mm per year. **(E)** Percentage of county's land area affected by VLM < -4 mm per year. **(F)** Percentage of county's land area affected by VLM < -5 mm per year. Note that this figure considers the upper bound (VLM - standard deviation), see Figs. 3 and S6 for the median (VLM) and lower bounds (VLM + standard deviation). The black and gray shape outline in Figs. 3A to F defines the extent of each county. The light grey counties in Figs. S7A to F show counties without any SAR pixels exposed to the different subsidence rates. Table 1 summarizes the number of counties, total land area in km^2, population, and properties exposed to subsidence. A summary table of all counties and land area exposure is provided in tables S5.

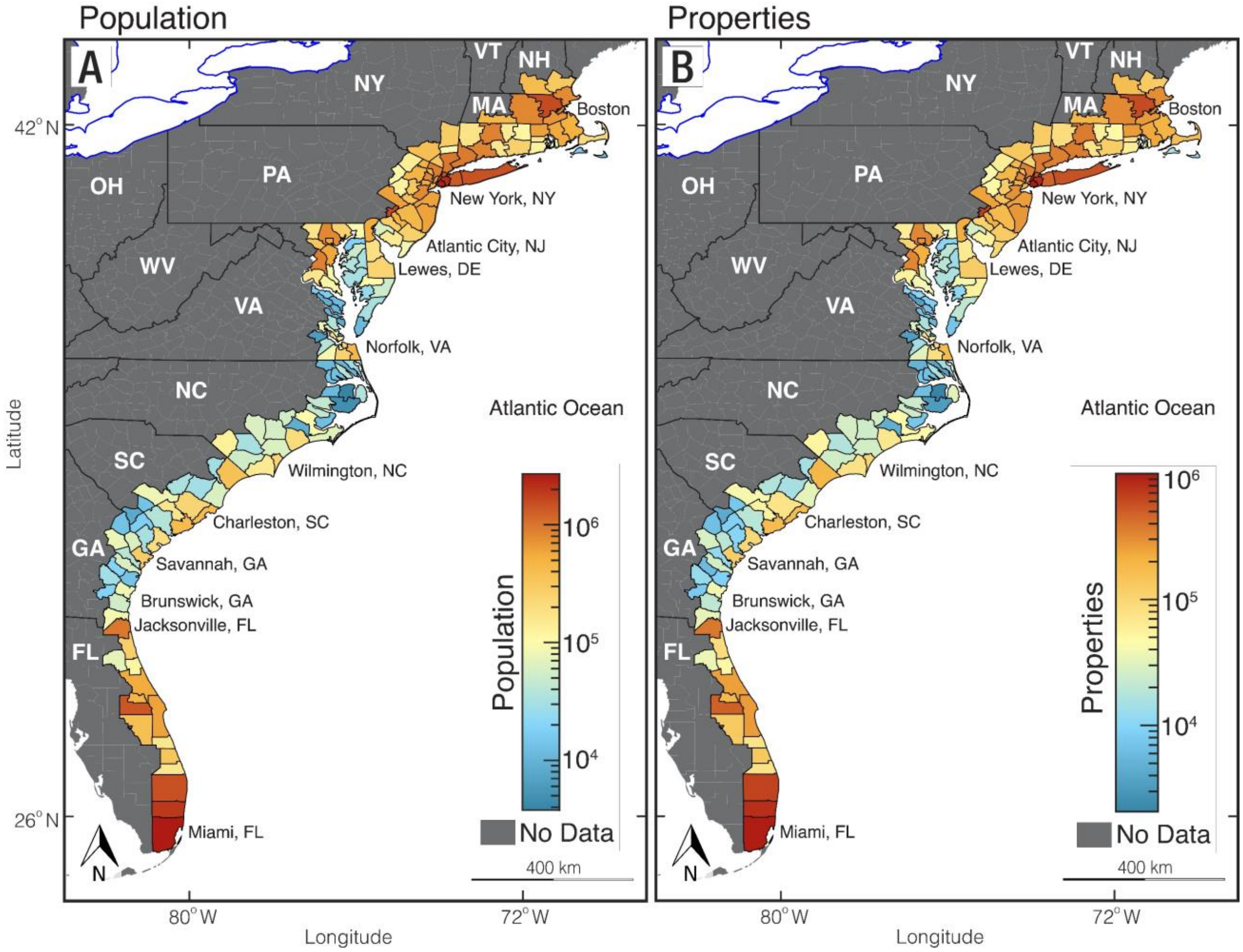

Fig. S8. Population and Properties on the U.S. east coast. (A) Distribution of population in 172 coastal counties. **(B)** Distribution of properties in 172 coastal counties. The black and gray shape outline defines the extent of each county. A summary table of all counties' population and properties is provided in table S2.

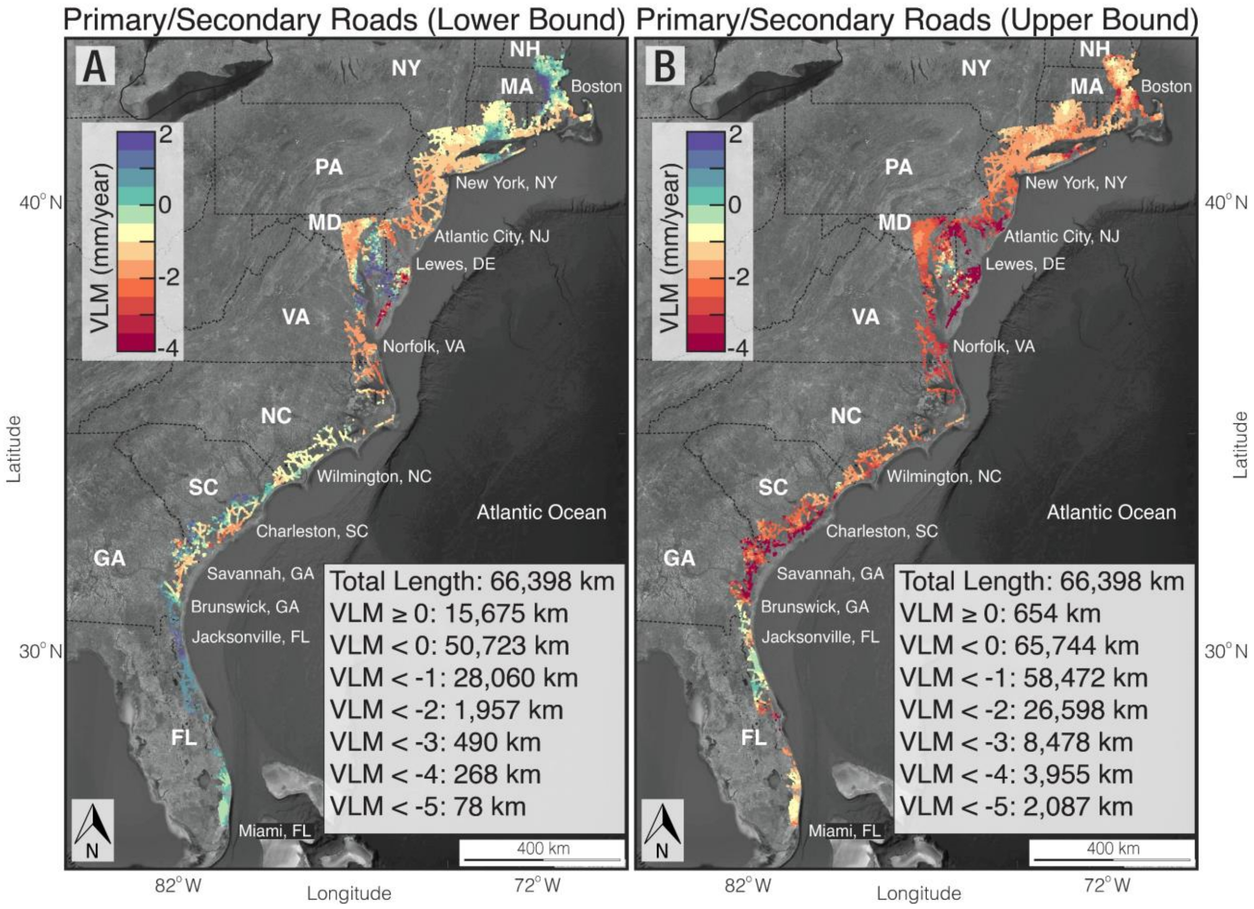

Fig. S9. Subsidence exposure for roads on the U.S. east coast. (A) Exposure to subsidence (lower bound) for primary/secondary roads. **(B)** Exposure to subsidence (upper bound) for primary/secondary roads. Background Images in (A) and (B) are from Google, Earthstar.

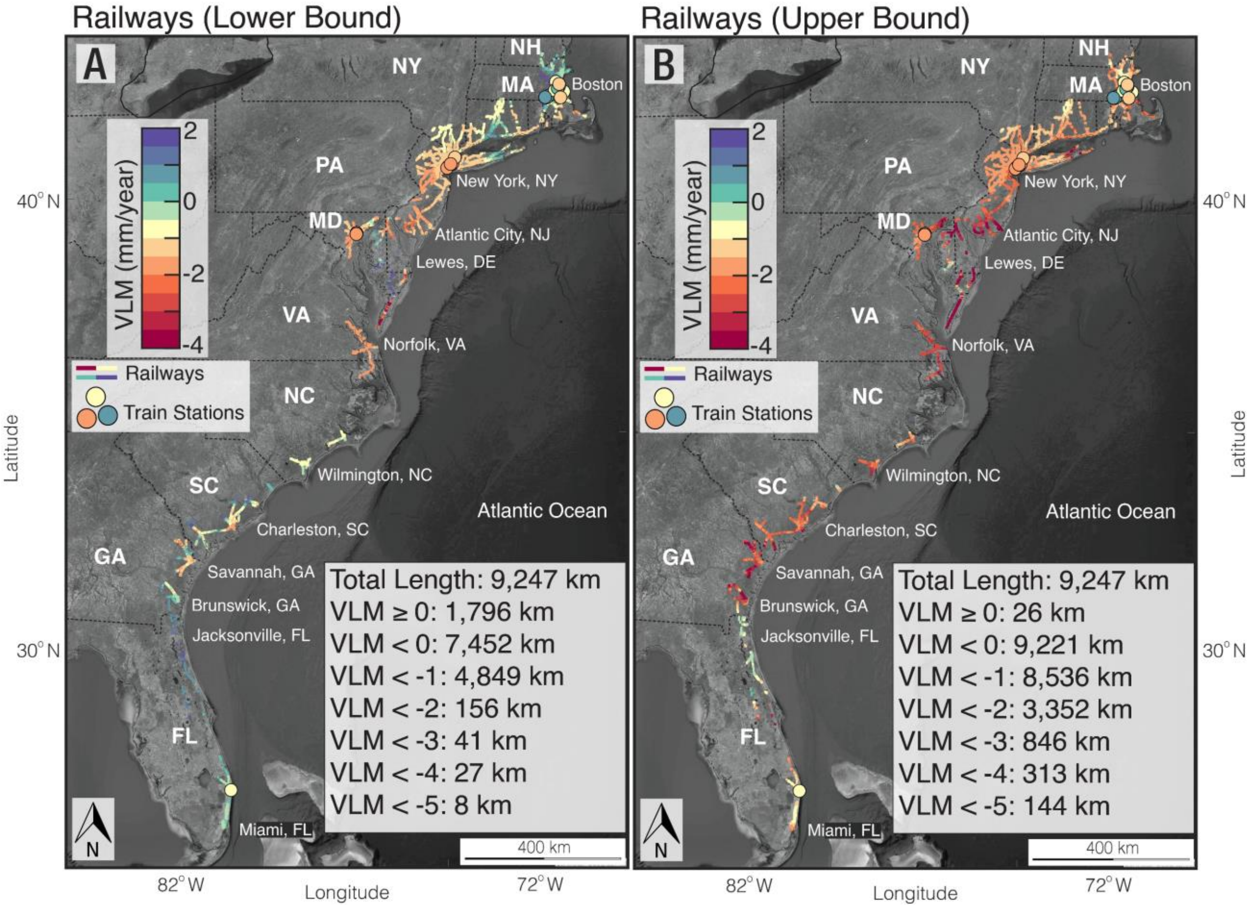

Fig. S10. Subsidence exposure for railways on the U.S. east coast. (A) Exposure to subsidence (lower bound) for railways. **(B)** Exposure to subsidence (upper bound) for railways. Background Images in (A) and (B) are from Google, Earthstar.

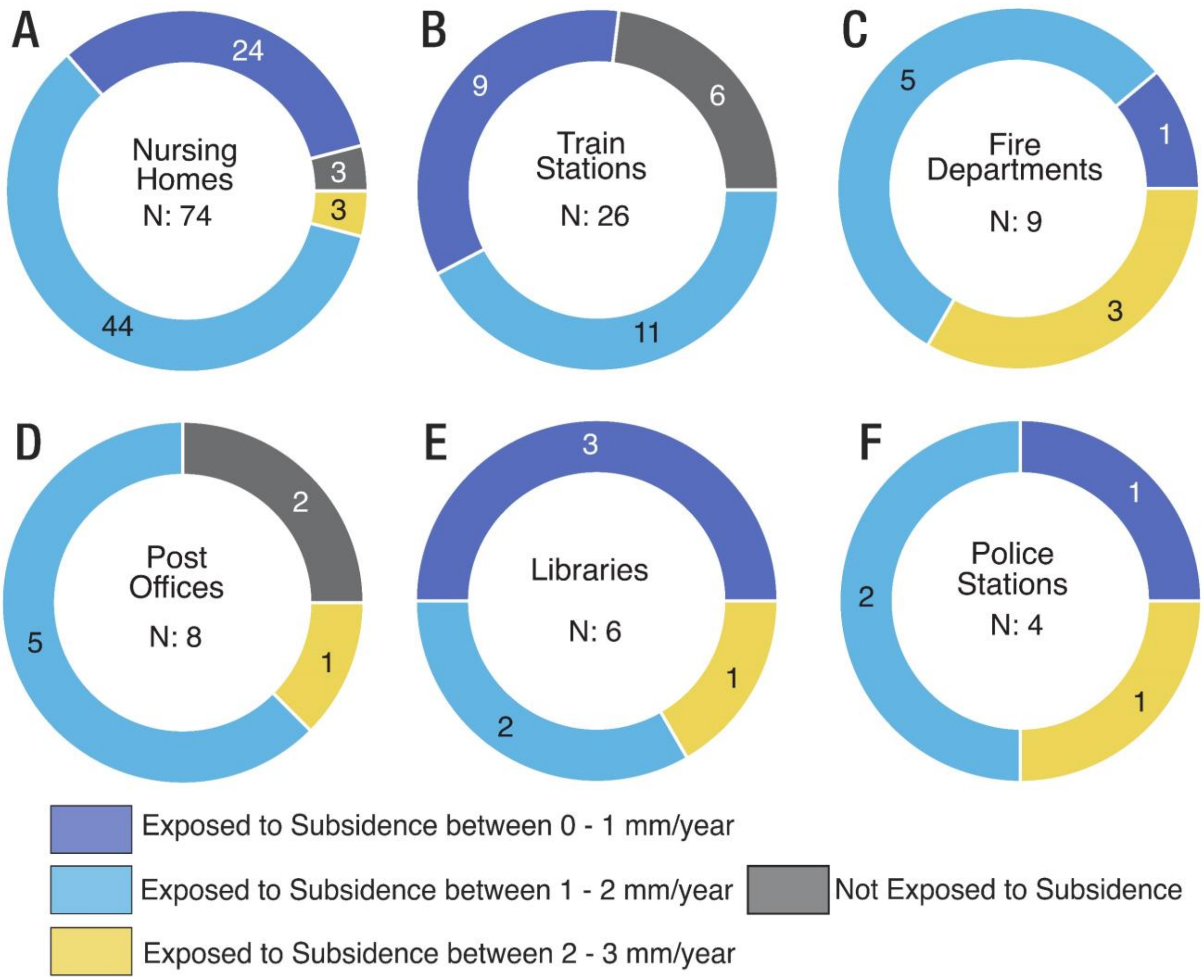

Fig. S11. Subsidence exposure for infrastructure facilities on the U.S. east coast. Pie chart showing subsidence exposure for **(A)** nursing homes, **(B)** train stations, **(C)** fire departments, **(D)** post offices, **(E)** libraries, and **(F)** police stations. N is the total number of infrastructure facilities. The number of infrastructure facility within each VLM category are shown in the pie chart. Not exposed to subsidence means VLM > 0 mm per year.

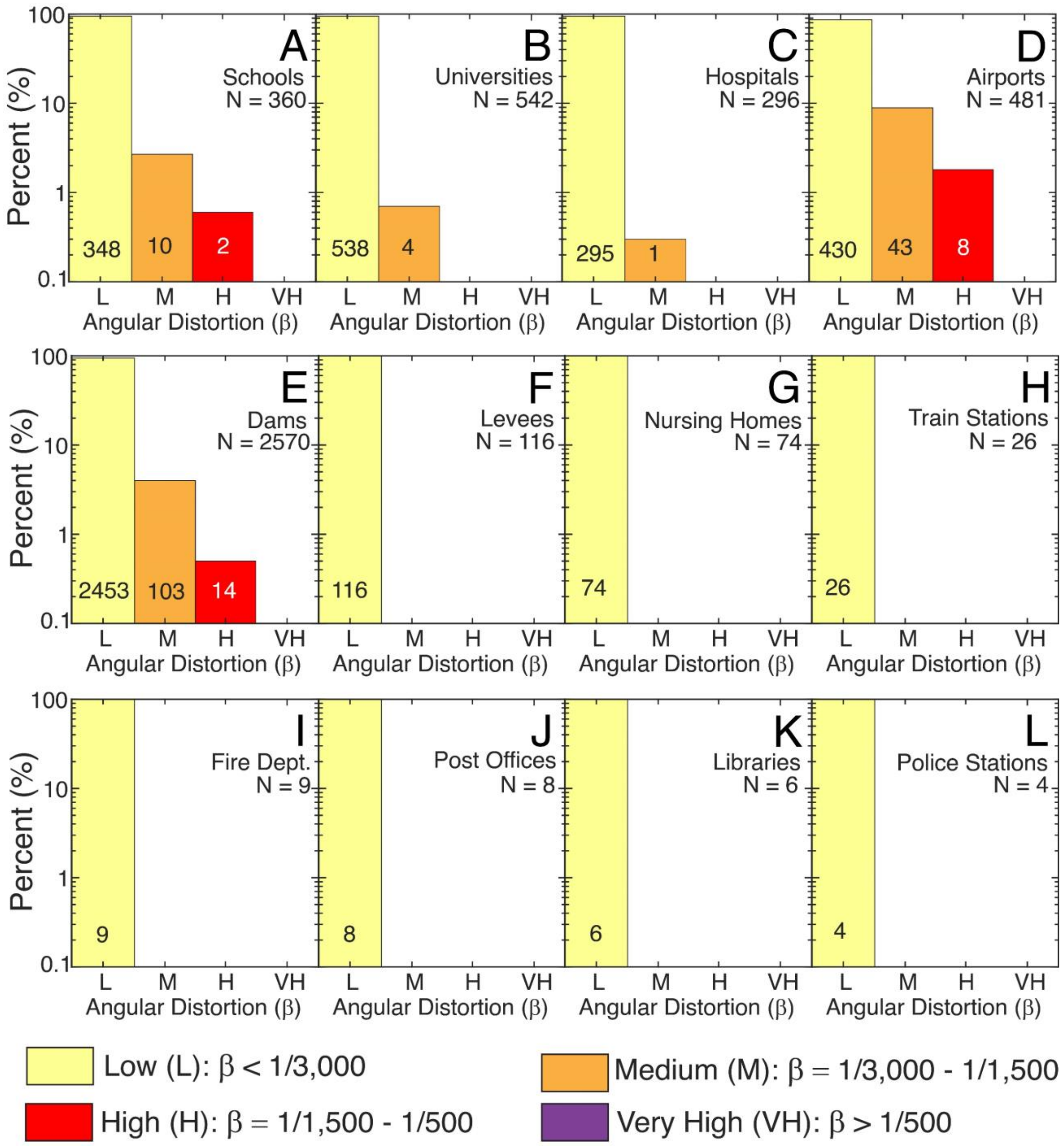

Fig. S12. Differential Subsidence Hazard Analysis for infrastructure facilities on the U.S. east coast. Histogram showing angular distortion category for **(A)** schools, **(B)** universities, **(C)** hospitals, **(D)** airports, **(E)** dams, **(F)** levees, **(G)** nursing homes, **(H)** train stations, **(I)** fire departments, **(J)** post offices, **(K)** libraries, and **(L)** police stations. N is the total number of infrastructure facilities. Note that the percent of infrastructure in each category uses a log scale to highlight some medium and high-hazard infrastructure.

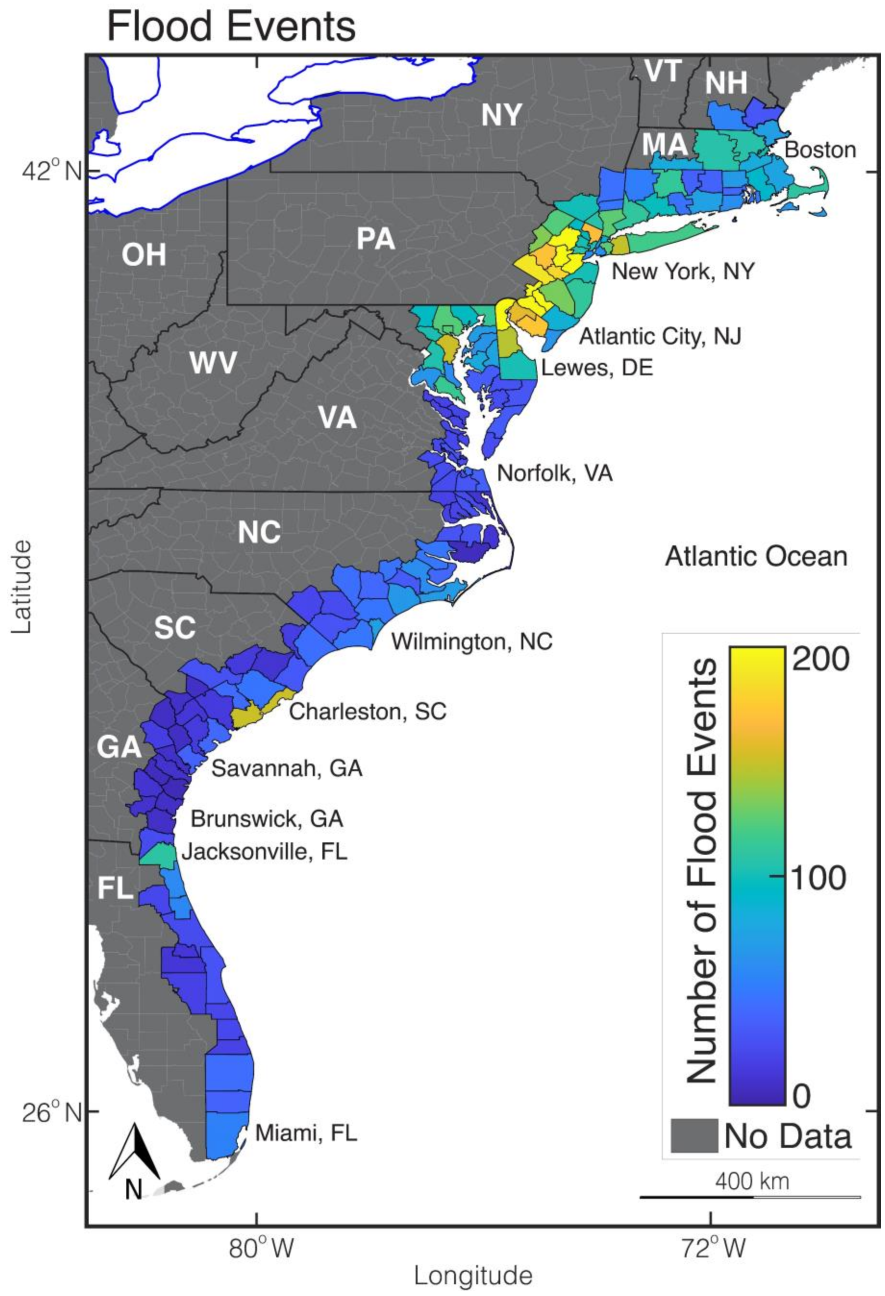

Fig. S13. Flood events in coastal counties on the U.S. east coast during 1996 – 2019. The black and gray shape outline defines the extent of each county. The flood events data is from the Federal Emergency Management Agency (FEMA) flood risk database (https://www.fema.gov/data-visualization/historical-flood-risk-and-costs). A summary table of all counties flood events is provided in table S2.

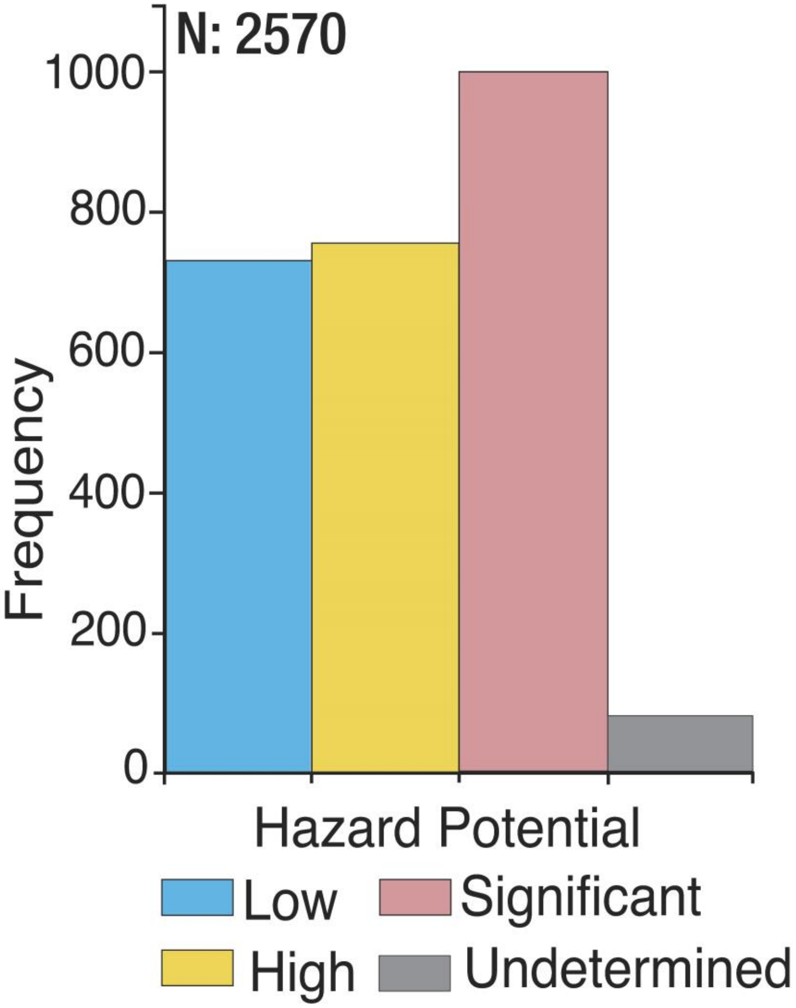

Fig. S14. Hazard potential Categories for dams on the U.S. east coast. Histogram showing the distribution of hazard potential for dams on the U.S. east coast analyzed in this study. N is the number of dams.

Table S1. Area of angular distortion category for twelve metropolitan cities on the U.S. east coast. State Codes – MA: Massachusetts, RI: Rhode Island, CT: Connecticut, NY: New York, NJ: New Jersey, MD: Maryland, VA: Virginia, NC: North Carolina, SC: South Carolina, GA: Georgia, and FL: Florida.

S/N	Cities	Area (km^2)			
		Low	Medium	High	Very High
1	Boston, MA	247.56	0.05	0	0
2	Providence, RI	41.78	0	0	0
3	New Haven, CT	51.63	0	0	0
4	New York, NY	561.01	0	0	0
5	Atlantic City, NJ	29.58	0	0	0
6	Baltimore, MD	228.48	0	0	0
7	Norfolk, VA	157.45	0	0	0
8	Wilmington, NC	541.34	0	0	0
9	Charleston, SC	935.07	0	0	0
10	Savannah, GA	1131.00	0	0	0
11	Jacksonville, FL	960.93	0	0	0
12	Miami, FL	78.37	0	0	0

Table S2. County-specific population, properties, and flood events for the U.S. east coast. State Codes NH: New Hampshire, MA: Massachusetts, RI: Rhode Island, CT: Connecticut, NY: New York, NJ: New Jersey, PA: Pennsylvania, MD: Maryland, DE: Delaware, VA: Virginia, NC: North Carolina, SC: South Carolina, GA: Georgia, and FL: Florida.

S/N	County Name	State Code	Population	Properties	Flood Events
1	Hillsborough	NH	418,735	166,053	56
2	Rockingham	NH	311,307	126,709	32
3	Dukes	MA	17,461	17,188	67
4	Nantucket	MA	11,376	11,618	70
5	Suffolk	MA	801,582	315,522	101
6	Worcester	MA	829,212	326,788	107
7	Bristol	MA	566,765	230,535	91
8	Norfolk	MA	709,409	270,359	82
9	Barnstable	MA	213,164	160,281	110
10	Hampden	MA	463,986	192,175	81
11	Essex	MA	791,263	306,754	73
12	Plymouth	MA	523,738	200,161	76
13	Middlesex	MA	1,609,379	612,004	107
14	Kent	RI	164,646	73,701	42
15	Newport	RI	81,836	41,796	51
16	Washington	RI	125,746	62,206	61
17	Providence	RI	636,547	264,835	69
18	Windham	CT	116,540	49,073	39
19	New London	CT	264,999	120,994	74
20	New Haven	CT	851,948	362,004	98
21	Litchfield	CT	179,610	87,550	53
22	Hartford	CT	889,226	374,249	113
23	Middlesex	CT	161,657	74,837	47
24	Fairfield	CT	942,426	361,221	111
25	Tolland	CT	150,600	57,963	39
26	Rockland	NY	326,225	104,057	106
27	Bronx	NY	1,401,142	511,896	87
28	Orange	NY	385,234	137,025	100
29	Queens	NY	2,225,821	835,127	125
30	Richmond	NY	475,327	176,656	62
31	Kings	NY	2,538,934	1,000,293	63
32	Nassau	NY	1,351,334	468,346	149
33	New York	NY	1,611,989	847,090	66
34	Suffolk	NY	1,474,273	569,985	118
35	Putnam	NY	98,532	38,224	49
36	Westchester	NY	965,802	370,821	125
37	Dutchess	NY	293,293	118,638	46

38	Morris	NJ	491,087	189,842	199
39	Cape May	NJ	91,546	98,309	66
40	Monmouth	NJ	618,381	258,410	101
41	Camden	NJ	506,809	204,943	223
42	Cumberland	NJ	147,008	55,834	173
43	Salem	NJ	62,451	27,417	159
44	Somerset	NJ	329,331	123,127	238
45	Union	NJ	555,394	199,489	108
46	Atlantic	NJ	262,945	126,647	86
47	Hunterdon	NJ	124,797	49,487	171
48	Mercer	NJ	367,239	143,169	182
49	Middlesex	NJ	822,736	294,800	205
50	Essex	NJ	800,501	312,954	101
51	Sussex	NJ	140,002	62,057	123
52	Hudson	NJ	671,666	270,335	103
53	Bergen	NJ	930,394	352,388	168
54	Ocean	NJ	614,237	278,052	112
55	Warren	NJ	105,624	44,925	134
56	Burlington	NJ	446,596	175,615	132
57	Gloucester	NJ	293,245	109,796	198
58	Passaic	NJ	500,382	175,966	94
59	Philadelphia	PA	1,578,487	670,171	234
60	Bucks	PA	627,987	245,956	189
61	Talbot	MD	36,972	19,577	80
62	Somerset	MD	25,453	11,130	27
63	Worcester	MD	52,403	55,749	32
64	Wicomico	MD	103,990	41,192	29
65	Anne Arundel	MD	582,777	212,562	151
66	Prince George's	MD	909,612	328,182	104
67	Baltimore	MD	826,017	335,622	123
68	Cecil	MD	103,419	41,103	114
69	Harford	MD	256,805	95,554	96
70	Baltimore	MD	586,131	296,685	123
71	Kent	MD	19,192	10,549	72
72	Charles	MD	164,436	54,963	66
73	Howard	MD	328,200	109,282	102
74	Caroline	MD	33,492	13,482	76
75	Dorchester	MD	31,853	16,554	36
76	Carroll	MD	169,092	62,406	95
77	Queen Anne's	MD	51,167	20,140	66
78	Calvert	MD	93,072	33,780	61
79	St. Mary's	MD	114,687	41,282	115
80	Kent	DE	183,643	65,338	146

81	New Castle	DE	561,531	217,511	254
82	Sussex	DE	241,635	123,036	102
83	Newport News	VA	179,062	76,198	27
84	Suffolk	VA	93,913	33,035	25
85	Williamsburg	VA	15,259	5,176	6
86	Virginia Beach	VA	451,231	177,879	37
87	Northumberland	VA	12,069	8,995	23
88	James City	VA	77,612	29,797	24
89	Lancaster	VA	10,618	7,402	22
90	Northampton	VA	11,673	7,301	27
91	Accomack	VA	32,238	21,002	35
92	Richmond	VA	9,071	3,850	21
93	Isle of Wight	VA	37,725	14,633	26
94	Surry	VA	6,385	3,444	26
95	Gloucester	VA	37,459	15,852	24
96	Norfolk	VA	242,803	95,018	45
97	Hampton	VA	135,464	59,566	25
98	Poquoson	VA	12,257	4,726	5
99	Portsmouth	VA	95,094	40,806	31
100	Chesapeake	VA	247,011	83,196	32
101	York	VA	69,199	26,849	33
102	Middlesex	VA	10,569	7,133	23
103	Westmoreland	VA	18,149	10,618	21
104	Mathews	VA	8,766	5,669	24
105	Columbus	NC	54,754	26,042	30
106	Lenoir	NC	55,720	27,437	47
107	Beaufort	NC	47,073	24,688	49
108	Pasquotank	NC	40,372	16,833	29
109	Duplin	NC	58,794	25,728	41
110	Brunswick	NC	149,039	77,482	49
111	Dare	NC	37,547	33,492	13
112	Washington	NC	11,485	6,491	31
113	Bladen	NC	32,911	17,718	25
114	Sampson	NC	63,382	27,234	45
115	Hyde	NC	4,843	3,347	7
116	New Hanover	NC	236,613	101,436	74
117	Craven	NC	101,233	45,002	61
118	Camden	NC	10,984	4,104	25
119	Gates	NC	11,464	5,208	21
120	Perquimans	NC	13,667	6,986	22
121	Robeson	NC	129,999	52,751	23
122	Carteret	NC	69,558	48,179	71
123	Onslow	NC	203,943	68,226	68

124	Pender	NC	64,671	26,724	48
125	Jones	NC	9,250	4,838	35
126	Pamlico	NC	12,715	7,534	42
127	Tyrrell	NC	3,774	2,068	29
128	Chowan	NC	13,815	7,289	20
129	Currituck	NC	29,052	14,453	13
130	Horry	SC	365,449	185,992	45
131	Dorchester	SC	165,737	55,186	43
132	Allendale	SC	8,331	4,486	10
133	Bamberg	SC	13,906	7,716	9
134	Beaufort	SC	195,656	93,023	42
135	Charleston	SC	417,981	169,984	150
136	Clarendon	SC	33,415	17,467	16
137	Marion	SC	30,158	14,953	18
138	Colleton	SC	37,481	19,901	16
139	Georgetown	SC	63,353	33,672	33
140	Williamsburg	SC	29,825	15,359	12
141	Jasper	SC	31,588	10,299	17
142	Berkeley	SC	235,987	73,372	48
143	Hampton	SC	18,053	9,140	14
144	Orangeburg	SC	85,343	42,504	29
145	Wayne	GA	30,023	12,199	12
146	Bryan	GA	40,755	11,842	10
147	Effingham	GA	65,765	19,884	8
148	Glynn	GA	85,568	40,716	9
149	Liberty	GA	63,004	26,731	11
150	Long	GA	20,171	6,039	7
151	McIntosh	GA	14,387	9,220	5
152	Camden	GA	55,388	21,114	9
153	Bulloch	GA	80,839	28,794	18
154	Screven	GA	14,012	6,739	8
155	Brantley	GA	19,202	8,086	11
156	Chatham	GA	289,463	119,323	39
157	Seminole	FL	474,171	181,307	14
158	Broward	FL	1,958,105	810,388	39
159	Flagler	FL	118,451	48,595	58
160	Osceola	FL	385,315	128,170	20
161	Miami-Dade	FL	2,707,303	989,435	54
162	Indian River	FL	162,518	76,346	23
163	St. Lucie	FL	337,186	137,029	24
164	Palm Beach	FL	1,507,600	664,594	44
165	Duval	FL	966,728	388,486	109
166	St. Johns	FL	278,715	89,830	60

167	Putnam	FL	74,815	37,337	23
168	Nassau	FL	91,113	35,009	26
169	Martin	FL	162,088	78,131	21
170	Volusia	FL	561,497	254,226	25
171	Orange	FL	1,404,396	487,839	14
172	Brevard	FL	608,459	269,864	30

Table S3. Lower bound county-specific percent area exposure to different levels of subsidence for the U.S. east coast. State Codes NH: New Hampshire, MA: Massachusetts, RI: Rhode Island, CT: Connecticut, NY: New York, NJ: New Jersey, PA: Pennsylvania, MD: Maryland, DE: Delaware, VA: Virginia, NC: North Carolina, SC: South Carolina, GA: Georgia, and FL: Florida.

S/N	County	State Code	Area Exposure					
			**VLM (<0 mm per year)**	**VLM (<-1 mm per year)**	**VLM (<-2 mm per year)**	**VLM (<-3 mm per year)**	**VLM (<-4 mm per year)**	**VLM (<-5 mm per year)**
1	Hillsborough	NH	9.317	0	0	0	0	0
2	Rockingham	NH	15.724	0	0	0	0	0
3	Dukes	MA	39.622	6.544	0	0	0	0
4	Nantucket	MA	27.834	0.636	0	0	0	0
5	Suffolk	MA	50.102	17.955	0	0	0	0
6	Worcester	MA	17.556	6.055	0	0	0	0
7	Bristol	MA	35.422	16.005	0	0	0	0
8	Norfolk	MA	1.826	0	0	0	0	0
9	Barnstable	MA	32.455	0.047	0	0	0	0
10	Hampden	MA	53.822	26.501	0	0	0	0
11	Essex	MA	4.635	0.004	0	0	0	0
12	Plymouth	MA	53.663	2.075	0	0	0	0
13	Middlesex	MA	54.343	44.856	0	0	0	0
14	Kent	RI	56.608	20.966	0	0	0	0
15	Newport	RI	3.853	0.014	0	0	0	0
16	Washington	RI	15.880	5.601	0	0	0	0
17	Providence	RI	75.714	22.921	0	0	0	0
18	Windham	CT	38.663	2.559	0	0	0	0
19	New London	CT	34.135	16.484	0	0	0	0
20	New Haven	CT	78.057	4.182	0	0	0	0
21	Litchfield	CT	44.364	0.094	0	0	0	0
22	Hartford	CT	48.272	26.425	0	0	0	0
23	Middlesex	CT	58.062	17.889	0	0	0	0
24	Fairfield	CT	47.257	29.174	0	0	0	0
25	Tolland	CT	71.386	69.005	0	0	0	0
26	Rockland	NY	33.141	0.595	0	0	0	0
27	Bronx	NY	69.553	69.553	0	0	0	0
28	Orange	NY	55.030	55.030	0	0	0	0
29	Queens	NY	51.927	51.927	0	0	0	0
30	Richmond	NY	80.620	79.704	0	0	0	0

31	Kings	NY	31.167	31.100	0	0	0	0
32	Nassau	NY	31.298	17.995	0	0	0	0
33	New York	NY	71.583	42.940	0	0	0	0
34	Suffolk	NY	67.225	33.642	0	0	0	0
35	Putnam	NY	3.009	0.826	0	0	0	0
36	Westchester	NY	56.568	53.252	0	0	0	0
37	Dutchess	NY	47.707	36.482	0	0	0	0
38	Morris	NJ	64.719	64.543	0	0	0	0
39	Cape May	NJ	29.556	29.550	0.233	0	0	0
40	Monmouth	NJ	63.491	61.646	14.673	0	0	0
41	Camden	NJ	36.391	34.691	2.861	0	0	0
42	Cumberland	NJ	68.163	68.094	0	0	0	0
43	Salem	NJ	71.574	71.574	0	0	0	0
44	Somerset	NJ	58.677	53.888	0.110	0	0	0
45	Union	NJ	24.469	24.258	0	0	0	0
46	Atlantic	NJ	49.870	49.870	0	0	0	0
47	Hunterdon	NJ	67.582	67.582	0	0	0	0
48	Mercer	NJ	66.325	66.309	0	0	0	0
49	Middlesex	NJ	20.800	4.087	0	0	0	0
50	Essex	NJ	62.394	62.394	0	0	0	0
51	Sussex	NJ	56.860	36.652	0	0	0	0
52	Hudson	NJ	63.952	62.972	0	0	0	0
53	Bergen	NJ	9.495	3.200	0	0	0	0
54	Ocean	NJ	55.567	52.965	0	0	0	0
55	Warren	NJ	14.860	14.856	3.270	0	0	0
56	Burlington	NJ	43.989	27.987	0	0	0	0
57	Gloucester	NJ	0.021	0.021	0	0	0	0
58	Passaic	NJ	0.795	0.795	0	0	0	0
59	Philadelphia	PA	28.619	16.242	0.213	0.008	0	0
60	Bucks	PA	7.445	2.513	0.489	0.076	0.003	0
61	Talbot	MD	47.759	33.199	21.183	10.598	3.708	0.905
62	Somerset	MD	10.630	5.194	1.745	0.232	0.055	0.009
63	Worcester	MD	65.124	64.725	2.829	0	0	0
64	Wicomico	MD	21.972	21.510	0.019	0	0	0
65	Anne Arundel	MD	52.865	52.566	0	0	0	0
66	Prince George's	MD	33.794	23.819	3.926	0	0	0
67	Baltimore	MD	42.025	23.214	0	0	0	0
68	Cecil	MD	78.433	78.433	0	0	0	0
69	Harford	MD	31.674	9.410	0.091	0	0	0

70	Baltimore	MD	0.407	0.407	0	0	0	0
71	Kent	MD	24.363	24.363	0	0	0	0
72	Charles	MD	17.618	6.767	1.684	0.136	0.018	0
73	Howard	MD	13.092	7.654	4.414	1.971	0.621	0.143
74	Caroline	MD	5.156	5.156	0	0	0	0
75	Dorchester	MD	34.302	14.032	0.257	0	0	0
76	Carroll	MD	34.861	13.181	0.013	0	0	0
77	Queen Anne's	MD	21.584	12.496	2.757	0	0	0
78	Calvert	MD	43.695	38.964	18.581	0.197	0.023	0.004
79	St. Mary's	MD	27.972	25.593	6.533	0	0	0
80	Kent	DE	22.469	9.183	1.606	0.201	0.019	0.003
81	New Castle	DE	38.028	38.028	2.846	0	0	0
82	Sussex	DE	28.220	28.220	0.036	0	0	0
83	Newport News	VA	64.130	64.130	13.043	0	0	0
84	Suffolk	VA	63.752	63.752	0.385	0	0	0
85	Williamsburg	VA	4.228	1.161	0.065	0	0	0
86	Virginia Beach	VA	44.071	44.071	3.285	0	0	0
87	Northumberland	VA	21.286	12.468	4.409	0	0	0
88	James City	VA	36.541	36.494	35.530	31.563	20.272	2.956
89	Lancaster	VA	24.120	20.467	17.223	12.871	8.256	3.623
90	Northampton	VA	0.384	0.064	0	0	0	0
91	Accomack	VA	18.260	18.260	2.842	0	0	0
92	Richmond	VA	4.722	4.722	0.491	0	0	0
93	Isle of Wight	VA	36.083	36.083	0	0	0	0
94	Surry	VA	68.144	68.144	0	0	0	0
95	Gloucester	VA	70.679	70.679	0.463	0	0	0
96	Norfolk	VA	40.414	40.414	0	0	0	0
97	Hampton	VA	43.891	43.891	0	0	0	0
98	Poquoson	VA	71.196	71.196	0.135	0	0	0
99	Portsmouth	VA	39.926	39.926	0.208	0	0	0
100	Chesapeake	VA	9.834	9.787	0.979	0	0	0
101	York	VA	3.599	2.904	2.308	0	0	0
102	Middlesex	VA	54.879	54.879	0.195	0	0	0
103	Westmoreland	VA	29.148	1.499	0	0	0	0
104	Mathews	VA	1.173	0	0	0	0	0
105	Columbus	NC	33.024	33.024	1.293	0	0	0
106	Lenoir	NC	8.800	0	0	0	0	0
107	Beaufort	NC	57.824	4.297	0	0	0	0
108	Pasquotank	NC	11.545	11.545	0	0	0	0

109	Duplin	NC	16.992	4.836	0	0	0	0
110	Brunswick	NC	9.828	0	0	0	0	0
111	Dare	NC	0.056	0	0	0	0	0
112	Washington	NC	13.390	5.817	0	0	0	0
113	Bladen	NC	37.409	0.115	0	0	0	0
114	Sampson	NC	22.140	0.358	0	0	0	0
115	Hyde	NC	22.996	22.996	4.869	0	0	0
116	New Hanover	NC	12.236	12.229	0.028	0	0	0
117	Craven	NC	57.827	57.240	0.007	0	0	0
118	Camden	NC	38.535	16.194	0	0	0	0
119	Gates	NC	63.775	0.006	0	0	0	0
120	Perquimans	NC	67.208	0.186	0	0	0	0
121	Robeson	NC	31.790	0	0	0	0	0
122	Carteret	NC	31.067	0.055	0	0	0	0
123	Onslow	NC	52.623	39.531	0.190	0	0	0
124	Pender	NC	23.864	23.756	0	0	0	0
125	Jones	NC	36.534	36.534	23.672	0	0	0
126	Pamlico	NC	13.490	4.219	0	0	0	0
127	Tyrrell	NC	9.629	1.896	0	0	0	0
128	Chowan	NC	0.004	0	0	0	0	0
129	Currituck	NC	52.999	16.587	4.758	0.398	0	0
130	Horry	SC	41.745	25.835	4.984	0.058	0	0
131	Dorchester	SC	0	0	0	0	0	0
132	Allendale	SC	38.108	14.065	0.312	0	0	0
133	Bamberg	SC	29.441	0.891	0	0	0	0
134	Beaufort	SC	0.766	0.025	0	0	0	0
135	Charleston	SC	32.593	6.267	0.039	0	0	0
136	Clarendon	SC	35.680	12.344	0	0	0	0
137	Marion	SC	19.659	5.389	0	0	0	0
138	Colleton	SC	10.879	1.714	0.003	0	0	0
139	Georgetown	SC	26.913	12.400	0.005	0	0	0
140	Williamsburg	SC	36.260	20.440	5.578	0.024	0	0
141	Jasper	SC	36.731	5.092	0	0	0	0
142	Berkeley	SC	24.818	22.928	0.201	0	0	0
143	Hampton	SC	1.873	1.661	0.029	0	0	0
144	Orangeburg	SC	62.166	48.654	2.898	0	0	0
145	Wayne	GA	0	0	0	0	0	0
146	Bryan	GA	10.129	6.195	1.202	0.009	0	0
147	Effingham	GA	1.414	0.825	0.071	0	0	0

148	Glynn	GA	0.105	0	0	0	0	0
149	Liberty	GA	45.441	23.936	0	0	0	0
150	Long	GA	0	0	0	0	0	0
151	McIntosh	GA	24.987	0	0	0	0	0
152	Camden	GA	0	0	0	0	0	0
153	Bulloch	GA	0	0	0	0	0	0
154	Screven	GA	3.050	0	0	0	0	0
155	Brantley	GA	0.077	0	0	0	0	0
156	Chatham	GA	0	0	0	0	0	0
157	Seminole	FL	20.060	0	0	0	0	0
158	Broward	FL	0	0	0	0	0	0
159	Flagler	FL	0	0	0	0	0	0
160	Osceola	FL	0	0	0	0	0	0
161	Miami-Dade	FL	3.055	0	0	0	0	0
162	Indian River	FL	0	0	0	0	0	0
163	St. Lucie	FL	0.012	0	0	0	0	0
164	Palm Beach	FL	6.944	0.031	0	0	0	0
165	Duval	FL	1.414	0.825	0.071	0	0	0
166	St. Johns	FL	0.105	0	0	0	0	0
167	Putnam	FL	45.441	23.936	0	0	0	0
168	Nassau	FL	0	0	0	0	0	0
169	Martin	FL	24.987	0	0	0	0	0
170	Volusia	FL	0	0	0	0	0	0
171	Orange	FL	0	0	0	0	0	0
172	Brevard	FL	3.050	0	0	0	0	0

Table S4. Medium county-specific percent area exposure to different levels of subsidence for the U.S. east coast. State Codes NH: New Hampshire, MA: Massachusetts, RI: Rhode Island, CT: Connecticut, NY: New York, NJ: New Jersey, PA: Pennsylvania, MD: Maryland, DE: Delaware, VA: Virginia, NC: North Carolina, SC: South Carolina, GA: Georgia, and FL: Florida.

S/N	County	State Code	Area Exposure					
			VLM (<0 mm per year)	VLM (<-1 mm per year)	VLM (<-2 mm per year)	VLM (<-3 mm per year)	VLM (<-4 mm per year)	VLM (<-5 mm per year)
1	Hillsborough	NH	18.034	2.368	0.009	0	0	0
2	Rockingham	NH	29.968	4.448	0.101	0	0	0
3	Dukes	MA	0	0	0	0	0	0
4	Nantucket	MA	39.661	39.562	0.887	0	0	0
5	Suffolk	MA	54.284	8.923	0.168	0	0	0
6	Worcester	MA	0	0	0	0	0	0
7	Bristol	MA	58.371	44.939	0.209	0.003	0	0
8	Norfolk	MA	43.013	15.929	1.466	0.067	0	0
9	Barnstable	MA	35.422	35.422	1.448	0	0	0
10	Hampden	MA	1.838	0.473	0	0	0	0
11	Essex	MA	57.350	5.287	0.022	0	0	0
12	Plymouth	MA	59.111	49.228	2.303	0.028	0	0
13	Middlesex	MA	19.913	0.494	0.002	0	0	0
14	Kent	RI	55.830	53.072	0	0	0	0
15	Newport	RI	54.343	54.343	0	0	0	0
16	Washington	RI	58.060	55.640	0	0	0	0
17	Providence	RI	12.273	1.208	0	0	0	0
18	Windham	CT	15.880	15.875	0	0	0	0
19	New London	CT	75.799	75.488	0	0	0	0
20	New Haven	CT	61.518	28.029	0.005	0	0	0
21	Litchfield	CT	34.135	34.132	0	0	0	0
22	Hartford	CT	78.439	28.949	0	0	0	0
23	Middlesex	CT	74.218	33.368	0	0	0	0
24	Fairfield	CT	48.393	46.430	0	0	0	0
25	Tolland	CT	58.631	41.008	0	0	0	0
26	Rockland	NY	47.257	47.257	0	0	0	0
27	Bronx	NY	71.386	71.386	0	0	0	0
28	Orange	NY	33.141	24.593	0	0	0	0
29	Queens	NY	69.553	69.553	0	0	0	0
30	Richmond	NY	55.030	55.030	0	0	0	0
31	Kings	NY	51.927	51.927	0	0	0	0
32	Nassau	NY	80.620	80.620	0	0	0	0
33	New York	NY	31.167	31.167	0	0	0	0
34	Suffolk	NY	34.223	31.364	0.469	0.001	0	0

35	Putnam	NY	71.583	71.583	0	0	0	0
36	Westchester	NY	67.225	67.225	0	0	0	0
37	Dutchess	NY	3.009	3.009	0	0	0	0
38	Morris	NJ	56.568	56.568	0	0	0	0
39	Cape May	NJ	47.707	47.707	15.608	0.005	0	0
40	Monmouth	NJ	64.719	64.719	0.814	0	0	0
41	Camden	NJ	29.556	29.556	17.732	0	0	0
42	Cumberland	NJ	63.491	63.491	63.385	2.213	0	0
43	Salem	NJ	36.405	36.405	31.574	0.073	0	0
44	Somerset	NJ	68.163	68.163	0	0	0	0
45	Union	NJ	71.574	71.574	0	0	0	0
46	Atlantic	NJ	58.695	58.695	52.573	6.044	0	0
47	Hunterdon	NJ	24.469	24.469	0	0	0	0
48	Mercer	NJ	49.870	49.870	0	0	0	0
49	Middlesex	NJ	67.582	67.582	0	0	0	0
50	Essex	NJ	66.325	66.325	0	0	0	0
51	Sussex	NJ	20.800	20.800	0	0	0	0
52	Hudson	NJ	62.394	62.394	0	0	0	0
53	Bergen	NJ	56.860	56.860	0	0	0	0
54	Ocean	NJ	63.952	63.952	12.964	0.191	0	0
55	Warren	NJ	9.495	9.495	0	0	0	0
56	Burlington	NJ	55.567	55.567	7.403	0.033	0	0
57	Gloucester	NJ	14.860	14.860	14.842	0	0	0
58	Passaic	NJ	43.989	43.989	0	0	0	0
59	Philadelphia	PA	0.021	0.021	0	0	0	0
60	Bucks	PA	0.795	0.795	0	0	0	0
61	Talbot	MD	39.870	30.060	4.800	0.191	0.011	0
62	Somerset	MD	23.129	10.999	2.996	0.678	0.082	0.014
63	Worcester	MD	61.497	53.348	38.249	29.237	18.870	9.759
64	Wicomico	MD	28.900	19.464	11.691	7.367	3.148	0.966
65	Anne Arundel	MD	65.124	65.124	24.211	0	0	0
66	Prince George's	MD	21.972	21.972	4.282	0	0	0
67	Baltimore	MD	52.865	52.865	0.217	0	0	0
68	Cecil	MD	44.164	42.560	22.154	6.137	0.150	0
69	Harford	MD	42.758	42.749	9.702	0.004	0	0
70	Baltimore	MD	78.433	78.433	0	0	0	0
71	Kent	MD	46.043	42.332	16.023	6.017	0.043	0
72	Charles	MD	0.407	0.407	0.185	0	0	0
73	Howard	MD	24.363	24.363	0	0	0	0
74	Caroline	MD	26.625	17.264	6.022	1.353	0.136	0.018
75	Dorchester	MD	23.148	12.845	7.681	4.799	2.577	1.048
76	Carroll	MD	5.156	5.156	1.170	0	0	0
77	Queen Anne's	MD	51.040	39.846	12.613	1.440	0.021	0

78	Calvert	MD	52.658	40.872	0.910	0	0	0
79	St. Mary's	MD	32.478	24.123	7.758	2.410	0.046	0
80	Kent	DE	45.995	45.995	45.752	14.843	5.477	2.192
81	New Castle	DE	28.307	28.097	23.721	9.091	0.009	0
82	Sussex	DE	62.066	55.558	44.238	24.365	10.528	2.511
83	Newport News	VA	38.028	38.028	38.028	0	0	0
84	Suffolk	VA	28.220	28.220	28.220	0	0	0
85	Williamsburg	VA	64.130	64.130	64.130	0	0	0
86	Virginia Beach	VA	63.752	63.752	63.752	0	0	0
87	Northumberland	VA	28.531	7.745	0.985	0.065	0	0
88	James City	VA	44.071	44.071	44.071	0	0	0
89	Lancaster	VA	45.080	28.499	11.171	2.749	0.286	0.011
90	Northampton	VA	36.546	36.546	36.541	36.489	35.024	30.458
91	Accomack	VA	40.286	34.752	27.714	21.854	16.962	11.809
92	Richmond	VA	1.242	0.743	0.192	0	0	0
93	Isle of Wight	VA	18.260	18.260	18.260	0	0	0
94	Surry	VA	4.722	4.722	4.722	0	0	0
95	Gloucester	VA	36.083	36.083	36.083	0	0	0
96	Norfolk	VA	68.144	68.144	68.144	0	0	0
97	Hampton	VA	70.679	70.679	70.679	0	0	0
98	Poquoson	VA	40.414	40.414	40.414	0	0	0
99	Portsmouth	VA	43.891	43.891	43.891	0	0	0
100	Chesapeake	VA	71.196	71.196	71.196	0	0	0
101	York	VA	39.926	39.926	39.926	0	0	0
102	Middlesex	VA	9.834	9.834	9.686	0.606	0	0
103	Westmoreland	VA	4.102	3.938	3.035	2.494	0.842	0
104	Mathews	VA	54.879	54.879	54.879	0	0	0
105	Columbus	NC	29.148	28.877	0	0	0	0
106	Lenoir	NC	0	0	0	0	0	0
107	Beaufort	NC	1.173	1.173	0	0	0	0
108	Pasquotank	NC	33.024	33.024	32.302	0	0	0
109	Duplin	NC	8.800	8.800	0	0	0	0
110	Brunswick	NC	60.310	56.854	0.085	0	0	0
111	Dare	NC	11.545	11.545	11.545	0	0	0
112	Washington	NC	17.701	17.556	2.371	0	0	0
113	Bladen	NC	9.828	9.828	0	0	0	0
114	Sampson	NC	0.056	0.056	0	0	0	0
115	Hyde	NC	13.390	13.390	0	0	0	0
116	New Hanover	NC	37.415	37.282	0	0	0	0
117	Craven	NC	22.140	18.733	0	0	0	0
118	Camden	NC	22.996	22.996	22.996	0	0	0
119	Gates	NC	12.236	12.236	12.066	0	0	0
120	Perquimans	NC	57.827	57.827	44.330	0	0	0

121	Robeson	NC	0	0	0	0	0	0
122	Carteret	NC	38.535	38.533	0	0	0	0
123	Onslow	NC	63.775	63.775	0	0	0	0
124	Pender	NC	67.224	67.052	0	0	0	0
125	Jones	NC	31.790	29.625	0	0	0	0
126	Pamlico	NC	31.067	26.651	0	0	0	0
127	Tyrrell	NC	52.686	52.686	22.449	0	0	0
128	Chowan	NC	23.864	23.864	13.462	0	0	0
129	Currituck	NC	36.534	36.534	36.534	0	0	0
130	Horry	SC	22.337	12.485	0	0	0	0
131	Dorchester	SC	25.758	16.072	2.415	0.019	0	0
132	Allendale	SC	2.626	0.004	0	0	0	0
133	Bamberg	SC	0	0	0	0	0	0
134	Beaufort	SC	58.413	56.743	42.446	10.326	2.933	0.613
135	Charleston	SC	45.387	42.413	25.302	12.339	2.144	0.125
136	Clarendon	SC	0	0	0	0	0	0
137	Marion	SC	0	0	0	0	0	0
138	Colleton	SC	50.772	45.512	6.350	0.564	0.003	0
139	Georgetown	SC	42.325	29.038	2.140	0	0	0
140	Williamsburg	SC	11.269	1.327	0.002	0	0	0
141	Jasper	SC	60.143	49.860	23.798	2.025	0.016	0
142	Berkeley	SC	47.786	38.148	5.718	0.123	0	0
143	Hampton	SC	47.389	20.662	1.116	0.028	0	0
144	Orangeburg	SC	0	0	0	0	0	0
145	Wayne	GA	23.378	14.930	3.763	0.633	0.016	0
146	Bryan	GA	34.264	28.494	18.688	2.362	0	0
147	Effingham	GA	58.827	47.475	21.646	7.584	1.598	0.080
148	Glynn	GA	57.545	46.009	22.029	2.778	0.011	0
149	Liberty	GA	24.938	24.898	23.406	15.568	1.844	0
150	Long	GA	1.877	1.873	1.584	0.872	0.073	0
151	McIntosh	GA	64.187	62.024	53.591	36.501	11.250	0
152	Camden	GA	20.446	0.007	0	0	0	0
153	Bulloch	GA	14.155	12.646	7.649	2.362	0.361	0.026
154	Screven	GA	1.528	1.479	0.961	0.243	0.036	0
155	Brantley	GA	16.199	2.201	0	0	0	0
156	Chatham	GA	46.082	45.893	18.749	0.238	0	0
157	Seminole	FL	29.290	0.026	0	0	0	0
158	Broward	FL	25.163	0	0	0	0	0
159	Flagler	FL	0.480	0	0	0	0	0
160	Osceola	FL	0.070	0.001	0	0	0	0
161	Miami-Dade	FL	6.171	0.681	0	0	0	0
162	Indian River	FL	26.274	7.674	0.019	0	0	0
163	St. Lucie	FL	40.544	0.053	0	0	0	0

164	Palm Beach	FL	38.665	0	0	0	0	0
165	Duval	FL	5.943	0	0	0	0	0
166	St. Johns	FL	7.168	0	0	0	0	0
167	Putnam	FL	0	0	0	0	0	0
168	Nassau	FL	0.118	0	0	0	0	0
169	Martin	FL	35.370	0	0	0	0	0
170	Volusia	FL	8.819	0	0	0	0	0
171	Orange	FL	17.056	3.526	0.016	0	0	0
172	Brevard	FL	40.059	31.464	11.873	0.314	0	0

Table S5. Upper bound county-specific percent area exposure to different levels of subsidence for the U.S. east coast. State Codes NH: New Hampshire, MA: Massachusetts, RI: Rhode Island, CT: Connecticut, NY: New York, NJ: New Jersey, PA: Pennsylvania, MD: Maryland, DE: Delaware, VA: Virginia, NC: North Carolina, SC: South Carolina, GA: Georgia, and FL: Florida.

S/N	County	State Code	Area Exposure					
			VLM (<0 mm per year)	VLM (<-1 mm per year)	VLM (<-2 mm per year)	VLM (<-3 mm per year)	VLM (<-4 mm per year)	VLM (<-5 mm per year)
1	Hillsborough	NH	18.082	13.486	1.692	0.057	0.002	0
2	Rockingham	NH	30.012	26.188	4.533	0.527	0.005	0
3	Dukes	MA	39.661	39.661	35.482	8.476	0	0
4	Nantucket	MA	58.380	52.750	7.838	0.542	0.075	0
5	Suffolk	MA	58.759	58.706	7.904	0.356	0.042	0.003
6	Worcester	MA	47.788	45.658	21.688	3.550	0.369	0.008
7	Bristol	MA	35.422	35.422	8.743	0	0	0
8	Norfolk	MA	1.838	1.832	0	0	0	0
9	Barnstable	MA	58.028	48.003	1.177	0.038	0.006	0
10	Hampden	MA	59.745	58.927	34.377	2.728	0.417	0.010
11	Essex	MA	35.018	27.590	7.325	0.098	0	0
12	Plymouth	MA	55.830	55.830	8.889	0	0	0
13	Middlesex	MA	54.343	54.343	0.026	0	0	0
14	Kent	RI	58.060	58.060	14.919	0	0	0
15	Newport	RI	12.517	12.517	3.826	0	0	0
16	Washington	RI	15.880	15.880	5.890	0	0	0
17	Providence	RI	75.799	75.799	15.667	0	0	0
18	Windham	CT	61.799	61.799	8.953	0	0	0
19	New London	CT	34.135	34.135	0.426	0	0	0
20	New Haven	CT	78.439	63.881	0.009	0	0	0
21	Litchfield	CT	74.223	74.223	22.852	0.033	0	0
22	Hartford	CT	48.393	48.393	0.608	0	0	0
23	Middlesex	CT	58.631	58.574	0.313	0	0	0
24	Fairfield	CT	47.257	47.257	1.589	0	0	0
25	Tolland	CT	71.386	71.386	1.956	0	0	0
26	Rockland	NY	33.141	31.748	0.031	0	0	0
27	Bronx	NY	69.553	69.553	10.108	0	0	0
28	Orange	NY	55.030	55.030	44.724	0	0	0

29	Queens	NY	51.927	51.927	15.208	0	0	0
30	Richmond	NY	80.620	80.620	15.282	0	0	0
31	Kings	NY	31.167	31.167	0	0	0	0
32	Nassau	NY	34.227	34.227	5.422	1.422	0.078	0.001
33	New York	NY	71.583	71.583	0.082	0	0	0
34	Suffolk	NY	67.225	67.225	1.311	0	0	0
35	Putnam	NY	3.009	3.009	0	0	0	0
36	Westchester	NY	56.568	56.568	1.157	0	0	0
37	Dutchess	NY	47.707	47.707	47.570	11.828	3.522	0.159
38	Morris	NJ	64.719	64.719	63.509	0	0	0
39	Cape May	NJ	29.556	29.556	29.538	0.257	0	0
40	Monmouth	NJ	63.491	63.491	63.491	54.576	10.620	0
41	Camden	NJ	36.405	36.405	36.405	13.168	0.146	0
42	Cumberland	NJ	68.163	68.163	2.554	0	0	0
43	Salem	NJ	71.574	71.574	1.095	0	0	0
44	Somerset	NJ	58.695	58.695	58.664	40.187	14.787	1.681
45	Union	NJ	24.469	24.469	1.857	0	0	0
46	Atlantic	NJ	49.870	49.870	8.699	0	0	0
47	Hunterdon	NJ	67.582	67.582	24.172	0	0	0
48	Mercer	NJ	66.325	66.325	0	0	0	0
49	Middlesex	NJ	20.800	20.800	0	0	0	0
50	Essex	NJ	62.394	62.394	2.985	0	0	0
51	Sussex	NJ	56.860	56.860	3.488	0	0	0
52	Hudson	NJ	63.952	63.952	54.099	10.795	1.987	0.013
53	Bergen	NJ	9.495	9.495	0.091	0	0	0
54	Ocean	NJ	55.567	55.567	49.825	3.420	1.096	0
55	Warren	NJ	14.860	14.860	14.860	3.401	0.005	0
56	Burlington	NJ	43.989	43.989	1.490	0	0	0
57	Gloucester	NJ	0.021	0.021	0.021	0	0	0
58	Passaic	NJ	0.795	0.795	0.107	0	0	0
59	Philadelphia	PA	48.912	44.157	32.461	3.210	0.614	0.097
60	Bucks	PA	41.814	29.471	17.328	9.177	4.020	0.790
61	Talbot	MD	67.198	63.401	55.876	46.110	33.691	24.518
62	Somerset	MD	50.415	41.402	31.775	24.216	16.225	10.256
63	Worcester	MD	65.124	65.124	54.391	0.064	0	0
64	Wicomico	MD	21.972	21.972	17.382	0.192	0	0
65	Anne Arundel	MD	52.865	52.865	46.012	0.031	0	0
66	Prince George's	MD	44.180	44.180	43.963	26.504	8.398	2.712

67	Baltimore	MD	42.758	42.758	42.758	12.281	0.746	0.004
68	Cecil	MD	78.433	78.433	56.284	0	0	0
69	Harford	MD	46.048	46.048	45.943	24.241	11.759	4.274
70	Baltimore	MD	0.407	0.407	0.407	0	0	0
71	Kent	MD	24.363	24.363	5.634	0	0	0
72	Charles	MD	29.888	27.074	16.478	6.241	1.619	0.313
73	Howard	MD	46.334	30.676	13.839	8.237	5.376	3.115
74	Caroline	MD	5.156	5.156	5.141	0.067	0	0
75	Dorchester	MD	52.041	51.847	45.556	13.768	4.931	1.040
76	Carroll	MD	53.235	53.235	52.730	0.072	0	0
77	Queen Anne's	MD	33.214	33.053	30.934	5.377	2.452	0.358
78	Calvert	MD	45.995	45.995	45.995	45.593	24.132	14.538
79	St. Mary's	MD	28.307	28.307	28.302	20.138	10.798	2.322
80	Kent	DE	63.140	62.979	62.611	62.189	59.050	47.659
81	New Castle	DE	38.028	38.028	38.028	0	0	0
82	Sussex	DE	28.220	28.220	28.220	0	0	0
83	Newport News	VA	64.130	64.130	64.130	0	0	0
84	Suffolk	VA	63.752	63.752	63.752	40.721	0	0
85	Williamsburg	VA	47.733	46.911	35.252	5.350	0	0
86	Virginia Beach	VA	44.071	44.071	44.071	0	0	0
87	Northumberland	VA	46.135	46.135	45.827	12.512	2.606	0.847
88	James City	VA	36.546	36.546	36.546	36.546	36.530	36.139
89	Lancaster	VA	45.107	43.882	41.359	38.496	33.904	27.129
90	Northampton	VA	2.305	2.305	1.511	0.557	0.064	0
91	Accomack	VA	18.260	18.260	18.260	0	0	0
92	Richmond	VA	4.722	4.722	4.722	0	0	0
93	Isle of Wight	VA	36.083	36.083	36.083	4.471	0	0
94	Surry	VA	68.144	68.144	68.144	0	0	0
95	Gloucester	VA	70.679	70.679	70.679	0	0	0
96	Norfolk	VA	40.414	40.414	40.414	0	0	0
97	Hampton	VA	43.891	43.891	43.891	0	0	0
98	Poquoson	VA	71.196	71.196	71.196	7.914	0	0
99	Portsmouth	VA	39.926	39.926	39.926	0	0	0
100	Chesapeake	VA	9.834	9.834	9.834	6.071	1.329	0
101	York	VA	4.102	4.102	4.074	3.260	2.625	1.542
102	Middlesex	VA	54.879	54.879	54.879	40.535	0.156	0
103	Westmoreland	VA	29.148	29.148	6.783	0	0	0
104	Mathews	VA	1.173	1.173	0.789	0	0	0

105	Columbus	NC	33.024	33.024	33.024	9.899	0	0
106	Lenoir	NC	8.800	8.800	0	0	0	0
107	Beaufort	NC	60.310	60.310	34.230	9.249	0	0
108	Pasquotank	NC	11.545	11.545	11.545	8.513	0	0
109	Duplin	NC	17.701	17.701	16.402	3.149	0	0
110	Brunswick	NC	9.828	9.828	2.762	0	0	0
111	Dare	NC	0.056	0.056	0	0	0	0
112	Washington	NC	13.390	13.390	3.371	0	0	0
113	Bladen	NC	37.415	37.415	30.397	3.448	0	0
114	Sampson	NC	22.140	22.140	3.298	0	0	0
115	Hyde	NC	22.996	22.996	22.996	8.637	0	0
116	New Hanover	NC	12.236	12.236	12.236	1.448	0	0
117	Craven	NC	57.827	57.827	57.686	4.256	0	0
118	Camden	NC	38.535	38.535	27.307	0	0	0
119	Gates	NC	63.775	63.775	0	0	0	0
120	Perquimans	NC	67.224	67.224	20.968	0	0	0
121	Robeson	NC	31.790	31.787	0.841	0	0	0
122	Carteret	NC	31.067	31.067	10.937	0	0	0
123	Onslow	NC	52.686	52.686	52.277	5.500	0.006	0
124	Pender	NC	23.864	23.864	23.064	2.501	0	0
125	Jones	NC	36.534	36.534	36.534	4.383	0	0
126	Pamlico	NC	22.337	21.054	3.047	0.038	0	0
127	Tyrrell	NC	25.838	25.838	23.313	7.066	0.354	0
128	Chowan	NC	2.831	2.831	1.536	0	0	0
129	Currituck	NC	58.413	58.413	57.283	45.799	31.985	11.097
130	Horry	SC	45.414	45.414	40.263	23.682	16.919	8.923
131	Dorchester	SC	0	0	0	0	0	0
132	Allendale	SC	50.883	50.883	50.255	11.150	1.261	0.062
133	Bamberg	SC	42.979	42.197	23.960	8.493	0.426	0
134	Beaufort	SC	18.850	18.845	4.635	0.146	0	0
135	Charleston	SC	62.240	62.199	57.584	40.005	17.210	2.450
136	Clarendon	SC	47.797	47.797	39.492	5.946	0.724	0.048
137	Marion	SC	51.996	51.996	32.373	0.526	0.076	0.037
138	Colleton	SC	24.522	22.628	16.853	8.147	2.638	0.557
139	Georgetown	SC	35.326	35.326	29.962	20.151	10.502	1
140	Williamsburg	SC	59.568	59.564	50.158	29.858	12	4
141	Jasper	SC	57.545	57.164	49.810	31.672	12.962	3.399
142	Berkeley	SC	24.938	24.938	24.679	23.064	18.452	12.815

143	Hampton	SC	1.877	1.877	1.859	1.573	1.111	0.396
144	Orangeburg	SC	64.453	63.821	60.764	54.180	44.960	33.272
145	Wayne	GA	59.540	15.474	0.644	0	0	0
146	Bryan	GA	14.551	14.548	12.912	9.788	5.035	1.798
147	Effingham	GA	1.531	1.531	1.298	0.971	0.547	0.204
148	Glynn	GA	17.622	17.374	9.078	0.505	0	0
149	Liberty	GA	46.082	46.082	44.105	19.495	5.151	0.066
150	Long	GA	34.877	29.142	3.084	0	0	0
151	McIntosh	GA	25.163	0.387	0	0	0	0
152	Camden	GA	66.644	0.010	0	0	0	0
153	Bulloch	GA	0.291	0.291	0.248	0.009	0	0
154	Screven	GA	6.180	4.237	0.982	0	0	0
155	Brantley	GA	26.274	26.274	22.051	4.706	0	0
156	Chatham	GA	40.544	40.544	13.404	0	0	0
157	Seminole	FL	38.665	27.547	0.016	0	0	0
158	Broward	FL	32.187	12.262	0	0	0	0
159	Flagler	FL	47.297	11.849	1.221	0	0	0
160	Osceola	FL	17.926	0.556	0	0	0	0
161	Miami-Dade	FL	35.370	32.042	0.089	0	0	0
162	Indian River	FL	29.921	8.068	0.169	0	0	0
163	St. Lucie	FL	19.507	19.277	15.276	2.031	0.033	0
164	Palm Beach	FL	40.079	40.057	37.898	30.867	15.079	0.945
165	Duval	FL	32.324	12.327	0.151	0	0	0
166	St. Johns	FL	47.465	11.831	1.250	0	0	0
167	Putnam	FL	0	0	0	0	0	0
168	Nassau	FL	17.938	0.575	0	0	0	0
169	Martin	FL	35.658	32.227	0.095	0	0	0
170	Volusia	FL	29.935	8.072	0.164	0	0	0
171	Orange	FL	19.681	19.455	15.436	2.015	0.023	0
172	Brevard	FL	40.169	40.150	37.969	30.930	15.097	0.945
